# Supplementary material for: Safety and tolerability of natural and synthetic cannabinoids in adults aged over 50 years: A systematic review and meta-analysis
Source: PLoS Med. 2021 Mar 29;18(3):e1003524. doi: 10.1371/journal.pmed.1003524 (PMC8007034; doi:10.1371/journal.pmed.1003524)
Supplement: S1 Text — Table A in S1 Text. Summary of randomised controlled trials of THC in older adults for studies with participants with mean age ≥50 years (N = 30) and studies with participants with age ≥50 years (N = 4, 13%; one ≥50 years; two ≥65 years; one ≥75 years). Table B in S1 Text. Summary of randomised controlled trials of THC:CBD combination in older adults for studies with participants with mean age ≥50 years (N = 26) and studies with participants recruited with age ≥50 years (N = 3, 11.5%; two ≥50 years; one ≥65 years). Table C in S1 Text. Summary estimates (incident rate ratio, IRR) from meta-analysis for the most commonly reported adverse events (AEs): IRR of participants experiencing AE with cannabinoid (THC) compared to placebo or active control condition. Table D in S1 Text. Summary estimates (incident rate ratio, IRR) from meta-analysis for the most commonly reported adverse events (AEs): IRR of participants experiencing AE with cannabinoid (THC:CBD combination) compared to placebo or active control condition. Table E in S1 Text. Characteristics of unpublished randomised trials of cannabinoids in older adults. Fig A in S1 Text. Funnel plots for all tolerability and safety outcomes: THC studies. (a) All-cause adverse events (AEs); (b) Treatment-related AEs; (c) All-cause serious adverse events (AAEs); (d) Treatment-related SAEs; (e) AE-related withdrawals; (f) deaths. Fig B in S1 Text. THC dose-related withdrawals in THC studies. Fig C in S1 Text. Forest plot of all-cause adverse events: THC studies (participants with ≥50 years of age). Numbers under the “Subjects (n)” column refer to analysed participants from the active and control intervention arms, respectively. Fig D in S1 Text. Forest plot of treatment-related serious adverse events: THC studies (participants with ≥50 years of age). Numbers under the “Subjects (n)” column refer to analysed participants from the active and control intervention arms, respectively. Fig E in S1 Text. Forest plot of all-cause serio [file pmed.1003524.s002.docx]

**S1 TEXT**

**Safety and tolerability of natural and synthetic cannabinoids in older adults: a systematic review and meta-analysis**

**METHODS in S1 Text**

**Search strategy**

Two categories of search terms were used. For subject groups we used: ‘Aged’ OR ‘frail’ OR ‘elderly’ OR ‘older’ OR ‘aging’ OR ‘ageing’ OR ‘geriatric’ OR ‘dementia’ OR ‘Parkinson’s’ OR Alzheimer’s’ OR ‘Huntington’s’ OR ‘demented’. For the intervention, we used: ‘Cannabinoids’ OR ‘cannabinoid’ OR ‘cannabinol’ OR ‘cannabidiol’ OR ‘tetrahydrocannabinol’ OR ‘THC’ OR ‘CBD’ OR ‘Sativex’ OR ‘nabilone’ OR ‘dronabinol’ OR ‘delta-9-tetrahydrocannabinol’ OR ‘delta-THC’ OR ‘medical cannabis’ OR ‘epidiolex’. The existing clinical query ‘Therapy/Broad’ was used in PubMed to select therapeutic studies. We identified additional studies from the reference lists of included studies and review articles. The search was complemented with information from ClinicalTrials.gov. We also contacted authors of the identified studies to clarify further appropriateness of inclusion if needed.

The search strategy identified 4132 citations (Pubmed n = 1305; OVID (Medline, EMBASE and Psychinfo) n = 2041; CINAHL n =786). We identified an additional 23 studies by examining the reference lists of review articles and searching for published studies through ClinicalTrials.gov. Adjustment for duplicates left 3688 citations. Of these, 3427 were excluded based on screening of title and abstract. 261 full text articles were retrieved and assessed based on the eligibility criteria. We identified a total of 74 studies that could be included for analysis based on the initial inclusion/exclusion criteria between January 1990 to 31^st^ Oct 2020. However, as this systematic review will focus on the analysis of randomised trials only, a further 28 records were excluded, leaving 46 studies to be included in our analysis.

Not all published cannabinoid trials provided safety information; we excluded two randomized controlled trials [1, 2], because they did not report or quantify adverse events and one study that used THCV [3] for the THC meta-analysis. For the THC:CBD meta-analysis, we included one study that used a combination of CBD and THCV [3] and also ran the meta-analysis after excluding this study.

**Quality assessment**

For assessing the overall quality of evidence, we used the GRADE (Grading of Recommendations Assessment, Development and Evaluation) criteria to rate risk of bias, publication bias, imprecision, inconsistency, indirectness, and magnitude of effect [4]. We have summarised the GRADE ratings of very low–, low-, moderate-, or high-quality evidence to reflect the extent to which we have confidence in the effect estimates are correct[5]. This was done by one reviewer (KM) and checked by a second reviewer (LV), and disagreements were resolved via discussion with a third reviewer (SB).

In this approach, evidence from randomised controlled trials (RCT) is initially rated as “high quality” but can be downgraded up to three levels to “moderate quality”, “low quality”, or “very low quality” based on five categories of limitations. A high-quality rating indicates that we are confident that the true effect is similar to the estimated effect; a very-low-quality rating indicates that the true effect is likely to be substantially different from the estimated effect. Limitations considered are the risk of bias (i.e., whether limitations in study design and execution would bias the effect estimate), indirectness of evidence (e.g., whether the adverse events (AEs) of cannabinoids had to be inferred from indirect evidence), imprecision (i.e., sample sizes, commonality of AE outcomes), and publication bias (i.e., selective publication of studies leading to a systematic bias in the effect estimate).

**RESULTS in S1 Text**

Our search identified 4132 hits (records) of which 261 were considered potentially relevant, based on title and abstract screening, and obtained as full-text studies. A total of 60 controlled studies (n= 6216 participants; from 1933.47 person-years of cannabinoid exposure) available as 46 published articles were included (Fig 1). One of the articles used all 3 interventions comparing each against placebo, so was considered as 3 studies[3]. Four articles studied both THC alone and THC:CBD combinations to compare with placebo[6-9] and one article studied both oral THC alone and CBD alone intervention to compare with placebo [10]. There was one article that had 2 phases, crossover challenge phase and RCT for same indication [11]. Another article studied the same intervention for two treatment groups compared with separate placebo groups [12]. Four articles used different doses of the same intervention compared with placebo group for each dose [3, 10, 13, 14].

Of the 6 completed but unpublished studies found on ClinicalTrials.gov, 2 were excluded as they were open label. Remaining 4 studies (3 RCTs and one withdrawal study) were not included in the meta-analyses as unpublished (Table E in S1 Text). The mean age for all these trials was ≥50 (we have now added a column for mean age), although the age cut-off for recruitment was ≥18 years.

The formulations used in THC studies were nabilone (6), dronabinol (marinol) (14), THC (3), THC extract spray (2) and Namisol (5). The combination THC:CBD trials used THC:CBD spray (18), cannabis extract (6) and THC:CBDV (2). The CBD studies used CBD preparations only.

The disease conditions investigated were classified into broader subgroups for analysis purpose as neurodegenerative (Alzheimer’s disease, Parkinson’s disease, Huntington’s disease. Amyotrophic lateral sclerosis), Multiple sclerosis, motor neuron disease, pain (neuropathic pain), cancer (cancer or chemotherapy related anorexia, pain or nausea/vomiting), other (type 2 diabetes mellitus, chronic obstructive pulmonary disease, fibromyalgia, raised intraocular pressure, cervical dystonia, healthy, pancreatitis, obstructive sleep apnoea and Levodopa induced dyskinesia in Parkinson’s disease).

**Risk of bias and certainty assessments**

Overall study quality GRADE (Grading of Recommendations Assessment, Development and Evaluation) [5] is reported in Table 1-3. A summary of the risk of bias of included studies for each category of CBM are presented in the Fig 15-19. Of 29 articles reporting THC studies, Van Amerongen et al [11] reported 2 different phases of treatment (challenge phase using cross-over design and treatment phase using parallel-arm design) in the same article and was evaluated for study quality for each phase separately, resulting in 30 evaluated studies. Of the 21 articles reporting on THC:CBD combination treatment, Fallon et al [15] reported two separate phase 3 parallel-arm RCTs, which were evaluated separately for study quality, resulting in 26 evaluated studies. Briefly, most RCTs reported adequate randomisation sequence generation and concealment, outcome objectiveness and masking of outcome assessors; however some studies had high risk of bias because of potential for unmasking of participants and study personnel and selective reporting of the safety outcome.

Most studies reported objective outcome assessments, however only 60% of studies reported that outcome assessors had been appropriately blinded. There was selective reporting in 45% of studies i.e., they did not report data for all the safety outcomes (AEs and SAEs) in the trial and reported them when they occurred more than once or were more common or when they had occurrence above a certain threshold (1%-10%). The authors judged 33 (55%) trials at low risk of bias, 20 (33%) trials at unclear risk of bias and 7 (12%) trials to have high risk of bias for safety outcome reporting (Fig 15-18). Overall, 36 (60%) evaluated trials were judged to be of moderate to high quality, of which 15 (42%) trials reported all AEs and SAEs. Of 29 evaluated articles using THC, 17 (59%) were of moderate quality, 9 (31%) low quality and 3 (10%) very low quality, while out of 21 evaluated articles using THC:CBD combination treatment, 13 (62%) were of moderate quality, 5 (24%) of low quality and 2 high quality (9.5%) and 1 very low quality (4.5%). Of the 3 evaluated articles using CBD, 1 (33%) was of moderate quality, while 2 (67%) were of low quality. 18 (62%) THC studies, 8 (38%) THC:CBD studies and 1 (33%) CBD study reported all AEs.

**THC studies**

There were in total 30 studies (15 crossover and 15 were RCTs) from 28 articles [6-11, 14, 16-36] (summarised in Table A in S1 Text). There were 1461 (analysed 1417) participants on medication and 1251 (analysed 1210) on placebo. The articles were published between 1991 to 2020. Studies were conducted in the UK (7), Canada (5), USA (5), Netherlands (6), Denmark (1), Germany (1), Austria (1) and Switzerland (2). The conditions for treatment were multiple sclerosis (5), dementia (6), cervical dystonia (1), cancer (6), chronic pancreatitis (1), intraocular pressure (1), fibromyalgia (1), diabetes (1), Huntington’s disease (HD) (1), Parkinson’s disease (PD) (2), obstructive sleep apnoea (1), healthy adults (1) and amyotrophic lateral sclerosis (1). All the studies compared with placebo except for four studies, which compared with active treatments such as amitriptyline [23], Prochlorperazine [16], diazepam [33] and Megestrol acetate [19]. Mean age ranged from 50-87 years. The proportion of male participants ranged from 0-100%.

All cause AEs (k=21): Pooled IRR for all RCTs was 1.42 (95% CI,1.12-1.79), with 1.40 (95% CI, 1.02- 1.92) for parallel-arm and 1.46 (95% CI, 0.98- 2.169) for crossover studies. Egger’s test indicated no significant effect of publication or other selection bias t=0.85, df=19, p=0.40). Trim and fill method identified one missing study with the estimated effect still remaining significant after their inclusion (1.39, 95% CI:1.10- 1.76; p=0., *k*=22) (Fig A in S1 Text). Results of dependent meta-analysis of all cause AEs across all studies (IRR: 1.37, 95% CI:1.15- 1.6; *k*=21) were consistent with the result of independent meta-analysis.

Treatment related AEs (k=9): The IRR for all RCTs was 1.60 (95% CI, 1.26 -2.04), with 1.25 (95% CI, 0.81 -1.93) for parallel-arm and 1.78 (95% CI, 1.34 -2.37) for crossover studies. Egger’s test was not significant (t=0.69, df=7, p=0.51) and trim and fill method identified two missing studies with the estimated effect still remaining significant after their inclusion (1.53, 95% CI:1.18- 1.98; p=0., *k*=11) (Fig A in S1 Text).

All cause SAEs (k=27): Pooled IRR for all RCTs was 1.08 (95% CI, 0.80 -1.46), with 1.07 (95% CI, 0.77 -1.48 for parallel-arm and 1.18 (95% CI, 0.55 -2.51)for crossover RCTs. Egger’s test was not significant (t=-0.22, df=25, p=0.82) and Trim and fill method identified 10 missing studies with the estimated effect comparable but becoming significant after their inclusion (1.39, 95% CI:1.07- 1.81; p=0.01, *k*=37) (Fig A in S1 Text).

Results of dependent meta-analysis of all cause SAEs across all studies (IRR: 1.08, 95% CI:0.9- 1.26; *k*=27) were consistent with the result of independent meta-analysis.

Treatment related SAEs (k=23): Pooled IRR for all RCTs was 1.23 (95% CI, 0.56 -2.69), with 1.53 (95% CI, 0.48 -4.86) for parallel-arm and 1.02 (95% CI, 0.35 -2.96) crossover studies. Egger’s test indicated significant (t=-8.57, df=21, p= < .0001) publication bias. Trim and fill method identified 5 missing studies with the estimated effect comparable and non-significant after their inclusion (1.41, 95% CI:0.69- 2.88; p=0.35, *k*=28) (Fig A in S1 Text).

Results of dependent meta-analysis of treatment-related SAEs across all studies (IRR: 1.23, 95% CI:0.97- 1.49; *k*=23) were consistent with the result of independent meta-analysis.

Withdrawals (k=): Pooled RR for all studies was 1.18 (95% CI,0.89 -1.57), with 1.16 (95% CI,0.79 -1.71) for parallel-arm and 1.36 (95% CI, 0.65 -2.72) for crossover RCTs. Egger’s test was not significant (t=1.06, df=25, p=0.3) and trim and fill method did not identify any missing study (Fig A in S1 Text).

Results of dependent meta-analysis of AE-related withdrawals across all studies (RR: 1.17, 95% CI:0.88- 1.46; *k*=27)) were consistent with the result of independent meta-analysis.

All deaths (k=30): The IRR for all studies was 1.09 (95% CI, 0.75 -1.59), with 1.12 (95% CI, 0.74 -1.70) for parallel-arm and 0.92 (95% CI, 0.36 -2.38) for crossover studies. Egger’s test was not significant (t=0.39, df=28, p=0.69) and trim and fill method did not identify any missing study (Fig A in S1 Text).

Results of dependent meta-analysis of all deaths across all studies (RR: 1.09, 95% CI:0.88- 1.3; *k*=30) were consistent with the result of independent meta-analysis.

**THC:CBD combination studies**

There were in total 26 studies (5 crossover and 21 were RCTs) from 21 articles[3, 6-8, 12, 13, 15, 37-50] (summarised in Table B in S1 Text). Those on medications were 1965 (analysed 1940) and those on placebo 1887 (analysed 1863). The articles were published between 2003 to 2019. Twelve studies were conducted in UK, 3 were conducted in sites in multiple countries and one each in USA, Spain, Italy, Czech Republic, Germany and Switzerland. The conditions for treatment were multiple sclerosis (7), pain (5), cancer (7), diabetes (3), rheumatoid arthritis (1), PD (1), Motor Neuron Disease (1) and Chronic Obstructive Pulmonary Disease (COPD) (1). All the studies used placebo control and there was no active treatment comparator. Mean age ranged from 51-67 years for both treatment and placebo groups.

The proportion of male participants ranged from 0-80% for treatment group and 11-80% for placebo.

All cause AEs (k=16): Pooled IRR for all RCTs was 1.58 (95% CI, 1.26 -1.98), with 1.52 (95% CI,1.33- 1.72) for parallel-arm and 2.98 (95% CI, 0.75 -11.84) for crossover studies.

Egger’s test indicated no significant effect of publication or other selection bias (t=0.72, df=14, p=0.49)

Trim and fill method identified 4 missing studies with the estimated effect still remaining significant after their inclusion (1.43, 95% CI:1.07- 1.9; p=0.015, *k*=20) (Fig O in S1 Text). Results of dependent meta-analysis of all cause AEs across all studies (IRR: 1.65, 95% CI:1.22- 2.22; p=0.001, *k*=14) was consistent with the result of independent meta-analysis.

Treatment related AEs (k=9): Pooled IRR for all RCTs was 1.70 (95% CI,1.24 -2.33), with 1.67 (95% CI,1.21- 2.28) for parallel-arm and 4.73 (95% CI, 0.54 - 41.65) for crossover studies. Egger’s test was not significant (t=1.22, df=7, p=0.26). However, trim and fill method indicated 2 missing studies, with the estimated effect still remaining significant after their inclusion (1.62, 95% CI:1.20- 2.19; p=0.002, *k*=11) (Fig O in S1 Text).

Results of dependent meta-analysis of treatment-related AEs across all studies (IRR: 2.07, 95% CI:1.17-3.69, *k*=9) were consistent with the result of independent meta-analysis.

All cause SAEs (k=26): Pooled IRR for all RCTs was 1.17 (95% CI, 0.99 -1.39), with 1.18 (95% CI, 0.99 -1.40) for parallel-arm and 0.87 (95% CI, 0.15 -5.02) for crossover studies

Egger’s test was not significant (t=-0.40, df=24, p=0.69). However, trim and fill method indicated 4 missing studies, with the estimated effect becoming marginally significant after their inclusion (1.19, 95% CI: 1.0004- 1.41; p=0.05, *k*=30) (Fig O in S1 Text).

Results of dependent meta-analysis of all cause SAEs across all studies (IRR: 1.17, 95% CI:0.99- 1.37, *k*=26) were consistent with the result of independent meta-analysis.

Treatment related SAEs (k=21): Pooled IRR for all RCTs was 1.19 (95% CI, 0.88 -1.62), with 1.20 (95% CI, 0.88 -1.65) for parallel-arm and 0.87 (95% CI, 0.15 -5.02) for crossover studies. Egger’s test was not significant (t=-0.99, df=19, p=0.33). However, trim and fill method indicated 8 missing studies, with the estimated effect becoming significant after their inclusion (1.40, 95% CI:1.04- 1.89; p=0.03 *k*=29) (Fig O in S1 Text).

Results of dependent meta-analysis of treatment-related SAEs across all studies (IRR: 1.19, 95% CI:0.88- 1.62, *k*=21) were consistent with the result of independent meta-analysis.

Withdrawals (k=26): Pooled IRR for all RCTs was 1.40 (95% CI,1.08 -1.80), with 1.47 (95% CI,1.10 -1.98) for parallel-arm and 1.50 (95% CI, 0.33 -6.89) for crossover studies. Egger’s test was not significant (t=0.04, df=24, p=0.97) and trim and fill method did not identify any missing study (Fig O in S1 Text).

Results of dependent meta-analysis of AE-related withdrawals across all studies (RR: 1.58, 95% CI:1.09- 2.27, *k*=26) were consistent with the result of independent meta-analysis.

All deaths (k=26): Pooled IRR for all RCTs was IRR 1.14 (95% CI, 0.89 -1.46), with 1.15 (95% CI, 0.89 -1.48) for parallel-arm and 0.87 (95% CI, 0.15 -5.02) for crossover studies.

Egger’s test was not significant (t=-0.35, df=24, p=0.73). However, trim and fill method indicated 9 missing studies, with the estimated effect becoming significant after their inclusion (1.33, 95% CI:1.03- 1.7; p=0.026, *k*=35) (Fig O in S1 Text).

Results of dependent meta-analysis of all cause deaths across all studies (IRR: 1.14, 95% CI:0.89- 1.45; p=0.29, *k*=26) were consistent with the result of independent meta-analysis.

The meta-analysis was also run after excluding the study using THCV[3] and we found no change in direction or significance for any of the results (Fig I-N in S1 Text). Results of meta-analysis for individual AEs after excluding these studies was identical to the results including all studies (as shown in Table D in S1 Text) and hence not shown.

**CBD studies**

There were in total 4 studies (3 crossover and 1 parallel arm) from 3 articles [3, 10, 51]. Those on medications were 43 (analysed 40) and those on placebo 44 (analysed 41). The articles were published between 1991 to 2016. Two studies were conducted in UK, and one in USA. The conditions for treatment were Huntington’s disease, raised ocular pressure and Type 2 Diabetes mellitus (T2DM). The interventions used were oral CBD 20 mg, 40 mg, 200mg and 700mg per day. All the studies compared with placebo and there was no active treatment comparator. Mean age ranged from 53-59 years and the proportion of male participants ranged from 53-100%.

Safety outcome: One study reported no AEs [3]. In the other studies, number of patients with at least one AE were 18 in the treatment arm and 17 in the control arm. Total AEs of all causality were 489 for the treatment arm and 479 for control arm. One SAE was reported in control. There were 5 withdrawals, but none due to AE in either CBD or control arm. There were no deaths reported. For all cause AEs, Egger’s test was not significant (t=0.94, df=1, p=0.52) and trim and fill method did not identify any missing study (Fig X in S1 Text).

Common side effects reported in the treatment group were oral pain/discomfort (3), throat irritation (1), dizziness (1), bad taste (1), disturbed attention (1), increased diastolic BP (2), pharyngitis (2), feeling hot (1), decreased appetite (2). Oral pain/discomfort (2), dizziness (2), headache (2), hypoesthesia (2), increased Diastolic BP (2), and decreased appetite (2) was reported in the placebo group.

**Table A in S1 Text: Summary of randomised controlled trials of THC in older adults for studies with participants with mean age ≥50 years (N=30) and studies with participants with age ≥50 years (N=4, 13%; one ≥ 50 years; two ≥65 years; one ≥75 years)**

|  | Age stratification | N | Crossover, *N=15* | *Parallel-arm*, *N=15* |
| --- | --- | --- | --- | --- |
| Participants included (THC) | Mean age ≥ 50 years | 30 | 22 (9.5-26) | 24 (20-112.5) |
|  | Age ≥ 50 years | 4 | *13.5 (2-39) | NA |
| Participants included (control) | Mean age ≥ 50 years | 30 | 22 (9.5-26) | 25 (20-89.5) |
|  | Age ≥ 50 years | 4 | *13.5 (2-39) | NA |
| Participants analysed (THC) | Mean age ≥ 50 years | 30 | 22 (9.5-24) | 24 (18-112.5) |
|  | Age ≥ 50 years | 4 | *11.5 (2-38) | NA |
| Participants analysed (control) | Mean age ≥ 50 years | 30 | 22 (9.5-24) | 25 (16.5-89.5) |
|  | Age ≥ 50 years | 4 | *11.5 (3-38) | NA |
| Mean age in years (THC) | Mean age ≥ 50 years | 28 | 59.5 (54.5-75.5) | 60.4 (53.2-64.4) |
|  | Age ≥ 50 years | 4 | *75.4 (72-87) | NA |
| Mean age in years (control) | Mean age ≥ 50 years | 28 | 59.5 (54.5-75.5) | 59.5 (53.3-63.5) |
|  | Age ≥ 50 years | 4 | *73.4 (72-87) | NA |
| Male, % (THC) | Mean age ≥ 50 years | 30 | 62 (43-75.5) | 52 (39-59) |
|  | Age ≥ 50 years | 4 | *84.5 (50-100) | NA |
| Male, % (control) | Mean age ≥ 50 years | 30 | 62 (43-75.5) | 52 (39.5-67) |
|  | Age ≥ 50 years | 4 | *84.5 (50-100) | NA |
| Duration of THC treatment, weeks | Mean age ≥ 50 years | 30 | 2 (0.25-3) | 5 (2.8-7.1) |
|  | Age ≥ 50 years | 4 | *4 (0.4-6) | NA |
| Duration of control, weeks | Mean age ≥ 50 years | 30 | 2 (0.1-3) | 5 (2.8-8.7) |
|  | Age ≥ 50 years | 4 | *4 (0.1-6) | NA |
| Duration of study, weeks | Mean age ≥ 50 years | 30 | 8 (5-12) | 6 (3-9.5) |
|  | Age ≥ 50 years | 4 | *10 (4-14) | NA |
| Withdrawals total | Mean age ≥ 50 years | 29 | 2 (0-3.5) | 16.5 (4-27.8) |
|  | Age ≥ 50 years | 4 | *2.5 (0-12) | NA |
| THC, mg per day | Mean age ≥ 50 years | 30 | 5 (2.25-9) | 10 (4.8-25) |
|  | Age ≥ 50 years | 4 | *3.75 (1.6-6.5) | NA |
| Condition: |  |  |  |  |
|  |  |  |  |  |
| Alzheimer’s disease | Mean age ≥ 50 years | 30 | 0.20 (3) | 0.00 (0) |
|  | Age ≥ 50 years | 4 | 75.0 (3) | NA |
| ALS | Mean age ≥ 50 years | 30 | 0.07 (1) | 0.00 (0) |
|  | Age ≥ 50 years | 4 | 0.00 (0) | NA |
| Cancer | Mean age ≥ 50 years | 30 | 0.00 (0) | 0.33 (5) |
|  | Age ≥ 50 years | 4 | 0.00 (0) |  |
| Cervical dystonia | Mean age ≥ 50 years | 30 | 0.07 (1) | 0.00 (0) |
|  | Age ≥ 50 years | 4 | 0.00 (0) | NA |
| Dementia | Mean age ≥ 50 years | 30 | 0.13 (2) | 0.07 (1) |
|  | Age ≥ 50 years | 4 | 0.00 (0) | NA |
| Fibromyalgia | Mean age ≥ 50 years | 30 | 0.07 (1) | 0.00 (0) |
|  | Age ≥ 50 years | 4 | 0.00 (0) | NA |
| Huntington’s disease | Mean age ≥ 50 years | 30 | 0.07 (1) | 0.00 (0) |
|  | Age ≥ 50 years | 4 | 0.00 (0) | NA |
| Healthy older subjects | Mean age ≥ 50 years | 30 | 0.07 (1) | 0.00 (0) |
|  | Age ≥ 50 years | 4 | 25.0 (1) | NA |
| Intraocular pressure | Mean age ≥ 50 years | 30 | 0.07 (1) | 0.00 (0) |
|  | Age ≥ 50 years | 4 | 0.00 (0) | NA |
| Multiple sclerosis | Mean age ≥ 50 years | 30 | 0.13 (2) | 0.27 (4) |
|  | Age ≥ 50 years | 4 | 0.00 (0) | NA |
| OSA | Mean age ≥ 50 years | 30 | 0.00 (0) | 0.13 (2) |
|  | Age ≥ 50 years | 4 | 0.00 (0) | NA |
| Pain | Mean age ≥ 50 years | 30 | 0.00 (0) | 0.13 (2) |
|  | Age ≥ 50 years | 4 | 0.00 (0) | NA |
| Pancreatitis | Mean age ≥ 50 years | 30 | 0.07 (1) | 0.00 (0) |
|  | Age ≥ 50 years | 4 | 0.00 (0) | NA |
| Parkinson’s  disease | Mean age ≥ 50 years | 30 | 0.07 (1) | 0.07 (1) |
|  | Age ≥ 50 years | 4 | 0.00 (0) | NA |
| Active treatment: |  |  |  |  |
|  |  |  |  |  |
| Dronabinol  (Marinol) | Mean age ≥ 50 years | 30 | 0.33 (5) | 0.60 (9) |
|  | Age ≥ 50 years | 4 | 50.0 (2) | NA |
| Nabilone | Mean age ≥ 50 years | 30 | 0.27 (4) | 0.13 (2) |
|  | Age ≥ 50 years | 4 | 25.0 (1) | NA |
| Namisol | Mean age ≥ 50 years | 30 | 0.33 (5) | 0.07 (1) |
|  | Age ≥ 50 years | 4 | 25.0 (1) | NA |
| THC | Mean age ≥ 50 years | 30 | 0.07 (1) | 0.20 (3) |
|  | Age ≥ 50 years | 4 | 0.00 (0) | NA |

Data are median (IQR) or proportions (frequencies) unless otherwise stated. N is the number is non-missing values. THC = delta-9-tetrahydrocannabinol. CBD=cannabidiol. ALS=Amyotrophic lateral sclerosis. OSA=Obstructive sleep apnoea. *Data are median (range).

**Table B in S1 Text: Summary of randomised controlled trials of THC:CBD combination in older adults for studies with participants with mean age ≥50 years (N=26) and studies with participants recruited with age ≥50 years (N=3, 11.5%; two ≥50 years; one ≥65 years )**

|  | Age stratification | N | Crossover, *N=5* | *Parallel-arm*, *N=21* |
| --- | --- | --- | --- | --- |
| Participants included (THC:CBD) | Mean age ≥ 50 years | 26 | 18 (6-19) | 88 (31-128) |
|  | Age ≥ 50 years | 3 | *6 (5-19) | NA |
| Participants included (control) | Mean age ≥ 50 years | 26 | 18 (6-19) | 80 (30-118) |
|  | Age ≥ 50 years | 3 | *6 (5-19) | NA |
| Participants analysed (THC:CBD) | Mean age ≥ 50 years | 26 | 16 (5-17) | 87 (31-128) |
|  | Age ≥ 50 years | 3 | *5 (4-17) | NA |
| Participants analysed (control) | Mean age ≥ 50 years | 26 | 16 (5-17) | 80 (30-118) |
|  | Age ≥ 50 years | 3 | *5 (4-17) | NA |
| Mean age in years (THC:CBD) | Mean age ≥ 50 years | 26 | 58 (56-67) | 58.4 (51.9-59.4) |
|  | Age ≥ 50 years | 3 | *67 (58-67) | NA |
| Mean age in years (control) | Mean age ≥ 50 years | 26 | 58 (56-67) | 56 (52-59.6) |
|  | Age ≥ 50 years | 3 | *67 (62.5-67) | NA |
| Male, % (THC:CBD) | Mean age ≥ 50 years | 26 | 50 (49-63) | 50 (36-56) |
|  | Age ≥ 50 years | 3 | *63 (50-80) | NA |
| Male, % (control) | Mean age ≥ 50 years | 26 | 50 (49-63) | 48 (35-50) |
|  | Age ≥ 50 years | 3 | *63 (50-80) | NA |
| Duration of THC:CBD treatment, weeks | Mean age ≥ 50 years | 26 | 2 (0.1-4) | 5 (5-12) |
|  | Age ≥ 50 years | 3 | *0.1 (0.1-4) | NA |
| Duration of control, weeks | Mean age ≥ 50 years | 26 | 1 (0.1-4) | 5 (5-12) |
|  | Age ≥ 50 years | 3 | *0.1 (1-4) | NA |
| Duration of study, weeks | Mean age ≥ 50 years | 26 | 4 (1-10) | 9 (7-13) |
|  | Age ≥ 50 years | 3 | *1 (1-10) | NA |
| Withdrawals total | Mean age ≥ 50 years | 26 | 2 (1-2) | 20 (6-55) |
|  | Age ≥ 50 years | 3 | *1 (1-2) | NA |
| THC, mg per day | Mean age ≥ 50 years | 26 | 10.3 (10.2-21.6) | 20.8 (14.6-25) |
|  | Age ≥ 50 years | 3 | *10.2 (4.7-10.3) | NA |
| CBD, mg per day | Mean age ≥ 50 years | 26 | 9.5 (5.1-9.9) | 16.3 (12.5-23) |
|  | Age ≥ 50 years | 3 | *5.1 (4.4-9.5) | NA |
| THC by CBD ratio | Mean age ≥ 50 years | 26 | 1.08 (1.08-2.0) | 1.08 (1.08-1.09) |
|  | Age ≥ 50 years | 3 | *1.08 (1.07-2.0) | NA |
| Condition: |  |  |  |  |
|  |  |  |  |  |
| Cancer | Mean age ≥ 50 years | 26 | 0.20 (1) | 0.43 (9) |
|  | Age ≥ 50 years | 3 | 0.00 (0) | NA |
| COPD | Mean age ≥ 50 years | 26 | 0.20 (1) | 0.00 (0) |
|  | Age ≥ 50 years | 3 | 33.3 (1) | NA |
| Diabetes | Mean age ≥ 50 years | 26 | 0.00 (0) | 0.10 (2) |
|  | Age ≥ 50 years | 3 | 0.00 (0) | NA |
| Healthy Controls | Mean age ≥ 50 years | 26 | 0.20 (1) | 0.00 (0) |
|  | Age ≥ 50 years | 3 | 33.3 (1) | NA |
| Motor neurone  disease | Mean age ≥ 50 years | 26 | 0.00 (0) | 0.05 (1) |
|  | Age ≥ 50 years | 3 | 0.00 (0) | NA |
| Multiple  sclerosis | Mean age ≥ 50 years | 26 | 0.20 (1) | 0.29 (6) |
|  | Age ≥ 50 years | 3 | 0.00 (0) | NA |
| Pain | Mean age ≥ 50 years | 26 | 0.00 (0) | 0.14 (3) |
|  | Age ≥ 50 years | 3 | 0.00 (0) | NA |
| Parkinson’s  Disease | Mean age ≥ 50 years | 26 | 0.20 (1) | 0.00 (0) |
|  | Age ≥ 50 years | 3 | 33.3 (1) | NA |
| Active treatment: |  |  |  |  |
|  |  |  |  |  |
| Cannabis  extract | Mean age ≥ 50 years | 26 | 0.40 (2) | 0.19 (4) |
|  | Age ≥ 50 years | 3 | 33.3 (1) | NA |
| THC:CBDV | Mean age ≥ 50 years | 26 | 0.00 (0) | 0.10 (2) |
|  | Age ≥ 50 years | 3 | 0.00 (0) | NA |
| THC:CBD spray | Mean age ≥ 50 years | 26 | 0.60 (3) | 0.71 (15) |
|  | Age ≥ 50 years | 3 | 66.7 (2) | NA |

Data are median (IQR) or proportions (frequencies) unless stated otherwise. N is the number is non-missing values. THC = delta-9-tetrahydrocannabinol. CBD=cannabidiol. COPD=chronic obstructive pulmonary disease. *Data are median (range).

**Table C in S1 Text: Summary estimates (Incident rate ratio, IRR) from meta-analysis for the most commonly reported adverse events (AEs): IRR of participants experiencing AE with cannabinoid (THC) compared to placebo or active control condition**

| MedDRA* high-level grouping | Individual AEs | Summary IRR (95% CI) | I^2^, %^†^ | p | k^‡^ | QE ^§^ | QEp^\|\|^ |
| --- | --- | --- | --- | --- | --- | --- | --- |
| Gastrointestinal disorders | Nausea | 1.39 (0.89-2.19) | 2 | 0.15 | 22 | 12.79 | 0.92 |
|  | Vomiting | 1.67 (0.97-2.88) | 0 | 0.06 | 18 | 3.00 | 1.00 |
|  | Dry Mouth | 2.56 (1.59-4.14) | NA | 0.00 | 20 | 8.33 | 0.98 |
| Nervous System disorders | Dizziness/  Light-headedness | 2.25 (1.57-3.22) | 30 | 0.00 | 24 | 24.28 | 0.39 |
|  | Mobility/Balance/  Coordination problems | 1.41 (1.10-1.83) | 0 | 0.01 | 17 | 5.25 | 0.99 |
|  | Muscle weakness | 1.20 (0.83-1.74) | 0 | 0.33 | 18 | 2.94 | 1.00 |
|  | Headache/migraine | 1.65 (0.89-3.08) | 18 | 0.11 | 8 | 8.16 | 0.32 |
|  | Numbness/paraesthesia | 1.52 (0.87-2.67) | 0 | 0.14 | 4 | 0.69 | 0.87 |
| Psychiatric disorders | Sleep problems/ Insomnia | 1.09 (0.80-1.47) | 0 | 0.59 | 17 | 3.21 | 1.00 |
|  | Dissociative/Thinking/  Perception problems | 1.71 (0.87-3.39) | NA | 0.12 | 17 | 17.27 | 0.37 |
|  | Somnolence/Drowsiness | 1.49 (1.13-1.97) | 0 | 0.01 | 20 | 14.24 | 0.77 |
|  | Anxiety/Depression | 1.09 (0.81-1.48) | 0 | 0.57 | 13 | 1.98 | 1.00 |
|  | Concentration/attention problems | 1.76 (0.63-4.92) | 0 | 0.28 | 5 | 1.11 | 0.89 |
|  | Euphoria | 2.08 (1.00-4.32) | 0 | 0.05 | 6 | 1.48 | 0.92 |
| Cardiac disorders | Dyspnoea | 1.24 (0.55-2.79) | 0 | 0.61 | 16 | 1.40 | 1.00 |
|  | Palpitations | 1.22 (0.51-2.94) | 0 | 0.66 | 15 | 1.38 | 1.00 |
|  | Chest pain | 0.91 (0.35-2.35) | 0 | 0.84 | 16 | 1.14 | 1.00 |
| Vascular disorders | Hypotension | 1.12 (0.43-2.89) | 0 | 0.82 | 16 | 1.34 | 1.00 |
| Infections and infestations | Infection, unspecified | 0.93 (0.72-1.21) | 0 | 0.60 | 16 | 1.49 | 1.00 |
|  | Urinary tract infection | 0.80 (0.52-1.24) | 0 | 0.32 | 16 | 0.52 | 1.00 |
|  | Respiratory tract infection | 1.01 (0.42-2.43) | 0 | 0.98 | 16 | 4.39 | 1.00 |
| General disorders and administration  site conditions | Pain, non-specific | 0.94 (0.68-1.30) | 2 | 0.71 | 17 | 3.55 | 1.00 |
|  | Fatigue/tiredness | 1.12 (0.85-1.48) | 0 | 0.43 | 21 | 3.30 | 1.00 |
|  | Weakness/reduced mobility | 0.98 (0.64-1.52) | NA | 0.95 | 16 | 3.47 | 1.00 |
| Blood and Lymphatic System disorders | Anaemia | 1.07 (0.54-2.13) | 0 | 0.85 | 16 | 1.02 | 1.00 |
| Ear and Labyrinth disorders | Vertigo | 1.12 (0.42-3.00) | 0 | 0.81 | 15 | 1.34 | 1.00 |
| Eye Disorders | Visual impairment/disturbances | 1.48 (0.78-2.80) | 0 | 0.23 | 15 | 2.31 | 1.00 |
| Injury, poisoning and procedural complications | Falls and injuries | 1.01 (0.75-1.37) | 0 | 0.93 | 15 | 2.65 | 1.00 |
| Investigations | Raised Gamma GT | 1.12 (0.47-2.69) | 0 | 0.80 | 16 | 2.87 | 1.00 |
| Metabolism and Nutrition disorders | Fluid retention | 0.90 (0.54-1.50) | 0 | 0.69 | 16 | 0.93 | 1.00 |
|  | Decreased Appetite | 0.92 (0.34-2.44) | 0 | 0.86 | 16 | 1.30 | 1.00 |
|  | Increased Appetite | 1.01 (0.39-2.66) | 0 | 0.98 | 16 | 0.94 | 1.00 |
| Musculoskeletal and connective tissue disorders | Spasm/stiffness | 1.02 (0.82-1.28) | 0 | 0.83 | 17 | 0.94 | 1.00 |
|  | Joint disorders | 0.85 (0.56-1.30) | 0 | 0.45 | 16 | 0.47 | 1.00 |
|  | Musculoskeletal pain | 1.07 (0.57-2.03) | NA | 0.83 | 18 | 9.30 | 0.93 |
| Reproductive system and breast disorders | Male impotence | 0.48 (0.24-0.94) | NA | 0.03 | 15 | 2.88 | 1.00 |
| Respiratory, thoracic, and mediastinal disorders | Nose Tenderness | 1.01 (0.37-2.75) | 0 | 0.98 | 15 | 0.94 | 1.00 |
| Skin and subcutaneous tissue disorders | Other skin problem | 0.69 (0.30-1.55) | 0 | 0.37 | 15 | 2.52 | 1.00 |
|  | Rash | 0.99 (0.42-2.35) | 0 | 0.98 | 15 | 0.95 | 1.00 |
|  | Pressure Sore | 0.67 (0.26-1.72) | 0 | 0.41 | 15 | 2.10 | 1.00 |
| Renal and Urinary disorder | Bladder symptoms | 1.05 (0.77-1.43) | 0 | 0.77 | 15 | 0.58 | 1.00 |

**Table D in S1 Text: Summary estimates (Incident rate ratio, IRR) from meta-analysis for the most commonly reported adverse events (AEs): IRR of participants experiencing AE with cannabinoid (THC:CBD combination) compared to placebo or active control condition**

| MedDRA* high-level grouping | Individual AEs | Summary IRR (95% CI) | I^2^, %^†^ | p | k^‡^ | QE ^§^ | QEp^\|\|^ |
| --- | --- | --- | --- | --- | --- | --- | --- |
| Gastrointestinal disorders | Nausea | 1.51 (1.20-1.9) | 0 | 0.00 | 20 | 6.34 | 1.00 |
|  | Vomiting | 1.54 (1.13-2.11) | 0 | 0.01 | 18 | 7.41 | 0.98 |
|  | Dry Mouth | 1.74 (1.10-2.75) | 41 | 0.02 | 16 | 22.74 | 0.09 |
| Nervous System disorders | Dizziness/  Light-headedness | 2.15 (1.54-3.01) | 58 | 0.00 | 21 | 42.46 | 0.00 |
|  | Headache | 1.02 (0.70-1.49) | 0 | 0.90 | 17 | 11.76 | 0.76 |
|  | Tremor/Incoordination | 1.51 (0.90-2.54) | 0 | 0.12 | 10 | 2.78 | 0.97 |
|  | Numbness/paraesthesia | 0.92 (0.51-1.67) | 0 | 0.80 | 9 | 0.36 | 1.00 |
|  | Altered taste | 1.83 (0.87-3.81) | 5 | 0.11 | 13 | 7.40 | 0.83 |
| Psychiatric disorders | Sleep problems | 1.07 (0.80-1.44) | 2 | 0.66 | 12 | 6.89 | 0.81 |
|  | Somnolence/Drowsiness | 2.55 (1.76-3.69) | 0 | 0.00 | 19 | 7.92 | 0.98 |
|  | Anxiety/Depression | 1.23 (0.78-1.94) | 0 | 0.38 | 11 | 13.52 | 0.20 |
|  | Attention/Concentration | 1.43 (0.59-3.42) | NA | 0.43 | 11 | 7.55 | 0.67 |
|  | Thinking/Perception/  Dissociative problems | 1.23 (0.78-1.94) | 0 | 0.38 | 11 | 13.52 | 0.20 |
|  | Disorientation | 4.08 (1.95-8.52) | 0 | 0.00 | 15 | 5.37 | 0.98 |
| Cardiac disorders | Dyspnoea | 1.32 (0.58-3.02) | 0 | 0.51 | 11 | 0.89 | 1.00 |
|  | Palpitation | 0.72 (0.23-2.26) | 0 | 0.57 | 10 | 2.60 | 0.98 |
|  | Dysrhythmia | 1.24 (0.35-4.37) | 0 | 0.74 | 9 | 1.11 | 1.00 |
| Vascular disorders | Hypotension | 1.29 (0.39-4.28) | 0 | 0.67 | 10 | 1.59 | 1.00 |
| Infections and infestations | Infection, unspecified | 0.96 (0.66-1.39) | 0 | 0.82 | 8 | 0.13 | 1.00 |
|  | Urinary tract infection | 1.21 (0.68-2.13) | 0 | 0.52 | 10 | 0.33 | 1.00 |
|  | Respiratory tract infection | 0.89 (0.44-1.79) | 0 | 0.75 | 11 | 2.28 | 0.99 |
| Renal and Urinary disorders | Renal and urinary symptoms | 1.07 (0.78-1.46) | 0 | 0.69 | 9 | 0.41 | 1.00 |
| General disorders and administration  site conditions | Pain, non-specific | 0.91 (0.49-1.70) | 51 | 0.77 | 14 | 18.62 | 0.14 |
|  | Fatigue | 1.78 (1.28-2.47) | 0 | 0.00 | 18 | 10.70 | 0.87 |
|  | Weakness/reduced mobility | 1.10 (0.84-1.43) | 0 | 0.49 | 14 | 4.95 | 0.98 |
| Blood and Lymphatic System disorders | Anaemia | 1.12 (0.69-1.81) | 0 | 0.66 | 14 | 2.17 | 1.00 |
| Ear and Labyrinth disorders | Vertigo | 2.51 (0.86-7.28) | 0 | 0.09 | 11 | 4.95 | 0.89 |
| Eye Disorders | Visual symptoms | 2.31 (1.06-5.05) | NA | 0.04 | 9 | 2.67 | 0.95 |
| Injury, poisoning and procedural complications | Fall | 1.71 (0.74-3.94) | 0 | 0.21 | 10 | 2.09 | 0.99 |
| Investigations | Reduced weight | 1.32 (0.65-2.64) | 0 | 0.44 | 13 | 1.83 | 1.00 |
|  | Raised Gamma GT | 1.11 (0.35-3.49) | 0 | 0.86 | 10 | 0.42 | 1.00 |
| Metabolism and Nutrition disorders | Decreased Appetite | 1.40 (0.84-2.34) | 0 | 0.20 | 14 | 2.00 | 1.00 |
|  | Anorexia | 0.88 (0.54-1.44) | 0 | 0.61 | 14 | 6.08 | 0.94 |
|  | Increased Appetite | 1.83 (0.62-5.42) | 0 | 0.27 | 10 | 2.52 | 0.98 |
| Musculoskeletal and connective tissue disorders | Spasm/stiffness | 0.94 (0.71-1.24) | 0 | 0.64 | 10 | 3.44 | 0.94 |
|  | Back pain | 0.84 (0.27-2.59) | 0 | 0.76 | 10 | 0.24 | 1.00 |
|  | Musculoskeletal pain | 0.72 (0.25-2.12) | 0 | 0.56 | 9 | 0.43 | 1.00 |
| Neoplasms, benign, malignant, and unspecified | Neoplasms Progression | 1.17 (0.89-1.54) | 10 | 0.26 | 15 | 7.09 | 0.93 |
| Respiratory, thoracic, and mediastinal disorders | Pharyngolaryngeal pain | 1.12 (0.47-2.66) | 0 | 0.79 | 10 | 0.26 | 1.00 |
| Skin and subcutaneous tissue disorders | Other skin problem | 0.71 (0.32-1.60) | 0 | 0.41 | 9 | 1.46 | 0.99 |
|  | Rash | 1.04 (0.41-2.62) | 0 | 0.94 | 10 | 0.18 | 1.00 |
|  | Pressure Sore | 0.62 (0.22-1.79) | 0 | 0.38 | 9 | 0.96 | 1.00 |
| Immune system disorder | Immune system disorder, unspecified | 1.82 (0.15-21.67) | 0 | 0.64 | 2 | 0.17 | 0.68 |

* , Medical Dictionary for Regulatory Activities (MedDRA) is the standardised international medical terminology used by regulatory authorities when reporting adverse events. ^†,^ I^2^= percent of total variability (heterogeneity plus sampling variability) attributed to heterogeneity among the true effects. ^‡^ , k= number of studies included in analysis. ^§^QE= test statistic for the test of heterogeneity. ^||^ , QEp= p value for the test of heterogeneity.

.

**TABLE E in S1 Text: Characteristics of unpublished randomised trials of cannabinoids in older adults**

| **Trial ID** | **Study design** | **Sample N** | **Indication** | **Mean age in years (SD)** | **Intervention/ Comparator** | **Intervention Duration** | **Daily dose** | **Withdrawals Intervention** | **Withdrawals Comparator** | **Number of patients with at least one AE (intervention/**  **comparator)** | **Most commonly reported AEs** | **Number of patients with at least one SAE (intervention/ comparator)** | **Most commonly reported SAEs** |
| --- | --- | --- | --- | --- | --- | --- | --- | --- | --- | --- | --- | --- | --- |
| **NCT01606176** | RCT (parallel group) | Total- 70  Intervention- 36  Comparator- 34 | Chronic Refractory Pain Due to Multiple Sclerosis | 54,58 (11.57) | Sativex/ Placebo | 3 weeks | Up to 120mg TCH/120mg CBD per day | 4 (2 due to AEs) | 3 (3 due to AEs) | 35/26 | 1. Dizziness  2. UTI  3. Headache | 0/1 | 1. Sepsis |
| **NCT00757822** | RCT (parallel group) | Total- 180  Intervention- 92  Comparator- 88 | Postoperative nausea and vomiting | 57.7 (12.7) | Dronabinol/  Ondansetron | 1 day | 5mg/day | 28 (0 due to AEs) | 31 (0 due to AEs) | 2/6 | 1. Swelling at incision site | 13/10 | Overnight hospitalization secondary to:  -1. urinary retention  -2. unanticipated extensive surgery  -3. excessive postoperative pain  4. Post-operative complication due to surgery |
| **NCT00710424** | RCT (parallel group) | Total- 297  Intervention- 149  Comparator- 148 | Pain due to diabetic neuropathy | 59.5 (10.54) | Sativex/ Placebo | 14 weeks | Up to 65 mg THC/60 mg CBD per day | 44 (30 due due to AEs) | 23 (12 due to AEs) | 120/101 | 1. Dizziness  2. Nausea  3. Vomiting | 14/12 | 1. Myocardial infarction  (all other SAEs reported for individual participants) |
| **NCT00713817** | Withdrawal RCT (parallel group) | Total- 19  Intervention- 10  Comparator- 9 | Neuropathic pain | 53.8 (7.98) | Sativex/ Placebo | 5 weeks | Up to 65 mg THC/60 mg CBD per day | 0 | 0 | 3/2 | 1. Headache  (all other AEs reported for individual participants) | 0/0 |  |

**References**

1. Chagas MHN, Zuardi AW, Tumas V, Pena-Pereira MA, Sobreira ET, Bergamaschi MM, et al. Effects of cannabidiol in the treatment of patients with Parkinson's disease: An exploratory double-blind trial. Journal of Psychopharmacology. 2014;28(11):1088-92. PubMed PMID: 600355457.

2. Selvarajah D, Gandhi R, Emery CJ, Tesfaye S. Randomized placebo-controlled double-blind clinical trial of cannabis-based medicinal product (Sativex) in painful diabetic neuropathy: depression is a major confounding factor. Diabetes care. 2010;33(1):128-30. Epub 2009/10/08. doi: 10.2337/dc09-1029. PubMed PMID: 19808912; PubMed Central PMCID: PMCPMC2797957.

3. Jadoon KA, Ratcliffe SH, Barrett DA, Thomas EL, Stott C, Bell JD, et al. Efficacy and Safety of Cannabidiol and Tetrahydrocannabivarin on Glycemic and Lipid Parameters in Patients With Type 2 Diabetes: A Randomized, Double-Blind, Placebo-Controlled, Parallel Group Pilot Study. Diabetes care. 2016;39(10):1777-86. Epub 2016/08/31. doi: 10.2337/dc16-0650. PubMed PMID: 27573936.

4. Guyatt GH, Oxman AD, Vist GE, Kunz R, Falck-Ytter Y, Alonso-Coello P, et al. GRADE: an emerging consensus on rating quality of evidence and strength of recommendations. BMJ (Clinical research ed). 2008;336(7650):924-6. Epub 2008/04/26. doi: 10.1136/bmj.39489.470347.AD. PubMed PMID: 18436948; PubMed Central PMCID: PMCPMC2335261 GRADE’s success has a positive influence on their academic career. Authors listed in the byline have received travel reimbursement and honorariums for presentations that included a review of GRADE’s approach to rating quality of evidence and grading recommendations. GHG acts as a consultant to UpToDate; his work includes helping UpToDate in their use of GRADE. HJS is documents editor and methodologist for the American Thoracic Society; one of his roles in these positions is helping implement the use of GRADE. He is supported by “The human factor, mobility and Marie Curie actions scientist reintegration European Commission grant: IGR 42192—GRADE.”.

5. Balshem H, Helfand M, Schünemann HJ, Oxman AD, Kunz R, Brozek J, et al. GRADE guidelines: 3. Rating the quality of evidence. Journal of clinical epidemiology. 2011;64(4):401-6. Epub 2011/01/07. doi: 10.1016/j.jclinepi.2010.07.015. PubMed PMID: 21208779.

6. Zajicek J, Fox P, Sanders H, Wright D, Vickery J, Nunn A, et al. Cannabinoids for treatment of spasticity and other symptoms related to multiple sclerosis (CAMS study): multicentre randomised placebo-controlled trial. The Lancet. 2003;362(9395):1517-26. doi: <https://doi.org/10.1016/S0140-6736(03)14738-1>.

7. Zajicek JP, Sanders HP, Wright DE, Vickery PJ, Ingram WM, Reilly SM, et al. Cannabinoids in multiple sclerosis (CAMS) study: safety and efficacy data for 12 months follow up. Journal of neurology, neurosurgery, and psychiatry. 2005;76(12):1664-9. Epub 2005/11/18. doi: 10.1136/jnnp.2005.070136. PubMed PMID: 16291891; PubMed Central PMCID: PMCPMC1739436.

8. Strasser F, Luftner D, Possinger K, Ernst G, Ruhstaller T, Meissner W, et al. Comparison of orally administered cannabis extract and delta-9-tetrahydrocannabinol in treating patients with cancer-related anorexia-cachexia syndrome: a multicenter, phase III, randomized, double-blind, placebo-controlled clinical trial from the Cannabis-In-Cachexia-Study-Group. Journal of clinical oncology : official journal of the American Society of Clinical Oncology. 2006;24(21):3394-400. Epub 2006/07/20. doi: 10.1200/jco.2005.05.1847. PubMed PMID: 16849753.

9. Johnson JR, Burnell-Nugent M, Lossignol D, Ganae-Motan ED, Potts R, Fallon MT. Multicenter, double-blind, randomized, placebo-controlled, parallel-group study of the efficacy, safety, and tolerability of THC:CBD extract and THC extract in patients with intractable cancer-related pain. Journal of pain and symptom management. 2010;39(2):167-79. Epub 2009/11/10. doi: 10.1016/j.jpainsymman.2009.06.008. PubMed PMID: 19896326.

10. Tomida I, Azuara-Blanco A, House H, Flint M, Pertwee RG, Robson PJ. Effect of sublingual application of cannabinoids on intraocular pressure: a pilot study. Journal of glaucoma. 2006;15(5):349-53. Epub 2006/09/22. doi: 10.1097/01.ijg.0000212260.04488.60. PubMed PMID: 16988594.

11. van Amerongen G, Kanhai K, Baakman AC, Heuberger J, Klaassen E, Beumer TL, et al. Effects on Spasticity and Neuropathic Pain of an Oral Formulation of Delta9-tetrahydrocannabinol in Patients WithProgressive Multiple Sclerosis. Clinical therapeutics. 2018;40(9):1467-82. Epub 2017/02/13. doi: 10.1016/j.clinthera.2017.01.016. PubMed PMID: 28189366.

12. Pickering EE, Semple SJ, Nazir MS, Murphy K, Snow TM, Cummin AR, et al. Cannabinoid effects on ventilation and breathlessness: a pilot study of efficacy and safety. Chronic respiratory disease. 2011;8(2):109-18. Epub 2011/03/26. doi: 10.1177/1479972310391283. PubMed PMID: 21436223.

13. Portenoy RK, Ganae-Motan ED, Allende S, Yanagihara R, Shaiova L, Weinstein S, et al. Nabiximols for opioid-treated cancer patients with poorly-controlled chronic pain: a randomized, placebo-controlled, graded-dose trial. The journal of pain : official journal of the American Pain Society. 2012;13(5):438-49. Epub 2012/04/10. doi: 10.1016/j.jpain.2012.01.003. PubMed PMID: 22483680.

14. Carley DW, Prasad B, Reid KJ, Malkani R, Attarian H, Abbott SM, et al. Pharmacotherapy of Apnea by Cannabimimetic Enhancement, the PACE Clinical Trial: Effects of Dronabinol in Obstructive Sleep Apnea. Sleep. 2018;41(1). doi: 10.1093/sleep/zsx184. PubMed PMID: 29121334; PubMed Central PMCID: PMCPMC5806568.

15. Fallon MT, Albert Lux E, McQuade R, Rossetti S, Sanchez R, Sun W, et al. Sativex oromucosal spray as adjunctive therapy in advanced cancer patients with chronic pain unalleviated by optimized opioid therapy: two double-blind, randomized, placebo-controlled phase 3 studies. Br J Pain. 2017;11(3):119-33. Epub 05/17. doi: 10.1177/2049463717710042. PubMed PMID: 28785408.

16. Lane M, Vogel CL, Ferguson J, Krasnow S, Saiers JL, Hamm J, et al. Dronabinol and prochlorperazine in combination for treatment of cancer chemotherapy-induced nausea and vomiting. Journal of pain and symptom management. 1991;6(6):352-9. Epub 1991/08/01. PubMed PMID: 1652611.

17. Volicer L, Stelly M, Morris J, McLaughlin J, Volicer BJ. Effects of dronabinol on anorexia and disturbed behavior in patients with Alzheimer's disease. International journal of geriatric psychiatry. 1997;12(9):913-9. Epub 1997/10/06. PubMed PMID: 9309469.

18. Sieradzan KA, Fox SH, Hill M, Dick JP, Crossman AR, Brotchie JM. Cannabinoids reduce levodopa-induced dyskinesia in Parkinson's disease: a pilot study. Neurology. 2001;57(11):2108-11. Epub 2001/12/12. PubMed PMID: 11739835.

19. Jatoi A, Windschitl HE, Loprinzi CL, Sloan JA, Dakhil SR, Mailliard JA, et al. Dronabinol versus megestrol acetate versus combination therapy for cancer-associated anorexia: a North Central Cancer Treatment Group study. Journal of clinical oncology : official journal of the American Society of Clinical Oncology. 2002;20(2):567-73. Epub 2002/01/12. doi: 10.1200/jco.2002.20.2.567. PubMed PMID: 11786587.

20. Svendsen KB, Jensen TS, Bach FW. Does the cannabinoid dronabinol reduce central pain in multiple sclerosis? Randomised double blind placebo controlled crossover trial. BMJ (Clinical research ed). 2004;329(7460):253. Epub 2004/07/20. doi: 10.1136/bmj.38149.566979.AE. PubMed PMID: 15258006; PubMed Central PMCID: PMCPMC498019.

21. Meiri E, Jhangiani H, Vredenburgh JJ, Barbato LM, Carter FJ, Yang HM, et al. Efficacy of dronabinol alone and in combination with ondansetron versus ondansetron alone for delayed chemotherapy-induced nausea and vomiting. Current medical research and opinion. 2007;23(3):533-43. Epub 2007/03/16. doi: 10.1185/030079907x167525. PubMed PMID: 17355735.

22. Curtis A, Mitchell I, Patel S, Ives N, Rickards H. A pilot study using nabilone for symptomatic treatment in Huntington's disease. Movement disorders : official journal of the Movement Disorder Society. 2009;24(15):2254-9. Epub 2009/10/22. doi: 10.1002/mds.22809. PubMed PMID: 19845035.

23. Ware MA, Fitzcharles MA, Joseph L, Shir Y. The effects of nabilone on sleep in fibromyalgia: results of a randomized controlled trial. Anesth Analg. 2010;110(2):604-10. Epub 2009/12/17. doi: 10.1213/ANE.0b013e3181c76f70. PubMed PMID: 20007734.

24. Weber M, Goldman B, Truniger S. Tetrahydrocannabinol (THC) for cramps in amyotrophic lateral sclerosis: a randomised, double-blind crossover trial. Journal of neurology, neurosurgery, and psychiatry. 2010;81(10):1135-40. Epub 2010/05/26. doi: 10.1136/jnnp.2009.200642. PubMed PMID: 20498181.

25. Brisbois TD, de Kock IH, Watanabe SM, Mirhosseini M, Lamoureux DC, Chasen M, et al. Delta-9-tetrahydrocannabinol may palliate altered chemosensory perception in cancer patients: results of a randomized, double-blind, placebo-controlled pilot trial. Annals of oncology : official journal of the European Society for Medical Oncology. 2011;22(9):2086-93. Epub 2011/02/24. doi: 10.1093/annonc/mdq727. PubMed PMID: 21343383.

26. Walther S, Schupbach B, Seifritz E, Homan P, Strik W. Randomized, controlled crossover trial of dronabinol, 2.5 mg, for agitation in 2 patients with dementia. Journal of clinical psychopharmacology. 2011;31(2):256-8. Epub 2011/03/03. doi: 10.1097/JCP.0b013e31820e861c. PubMed PMID: 21364345.

27. Zadikoff C, Wadia PM, Miyasaki J, Chen R, Lang AE, So J, et al. Cannabinoid, CB1 agonists in cervical dystonia: Failure in a phase IIa randomized controlled trial. Basal Ganglia. 2011;1(2):91-5. doi: <https://doi.org/10.1016/j.baga.2011.04.002>.

28. Toth C, Mawani S, Brady S, Chan C, Liu C, Mehina E, et al. An enriched-enrolment, randomized withdrawal, flexible-dose, double-blind, placebo-controlled, parallel assignment efficacy study of nabilone as adjuvant in the treatment of diabetic peripheral neuropathic pain. Pain. 2012;153(10):2073-82. Epub 2012/08/28. doi: 10.1016/j.pain.2012.06.024. PubMed PMID: 22921260.

29. Zajicek J, Ball S, Wright D, Vickery J, Nunn A, Miller D, et al. Effect of dronabinol on progression in progressive multiple sclerosis (CUPID): a randomised, placebo-controlled trial. The Lancet Neurology. 2013;12(9):857-65. Epub 2013/07/17. doi: 10.1016/s1474-4422(13)70159-5. PubMed PMID: 23856559; PubMed Central PMCID: PMCPMC3744749.

30. Ahmed AI, van den Elsen GA, Colbers A, van der Marck MA, Burger DM, Feuth TB, et al. Safety and pharmacokinetics of oral delta-9-tetrahydrocannabinol in healthy older subjects: a randomized controlled trial. European neuropsychopharmacology : the journal of the European College of Neuropsychopharmacology. 2014;24(9):1475-82. Epub 2014/07/19. doi: 10.1016/j.euroneuro.2014.06.007. PubMed PMID: 25035121.

31. Ahmed AI, van den Elsen GA, Colbers A, Kramers C, Burger DM, van der Marck MA, et al. Safety, pharmacodynamics, and pharmacokinetics of multiple oral doses of delta-9-tetrahydrocannabinol in older persons with dementia. Psychopharmacology. 2015;232(14):2587-95. Epub 2015/03/11. doi: 10.1007/s00213-015-3889-y. PubMed PMID: 25752889; PubMed Central PMCID: PMCPMC4480847.

32. van den Elsen GA, Ahmed AI, Verkes RJ, Kramers C, Feuth T, Rosenberg PB, et al. Tetrahydrocannabinol for neuropsychiatric symptoms in dementia: A randomized controlled trial. Neurology. 2015;84(23):2338-46. Epub 2015/05/15. doi: 10.1212/wnl.0000000000001675. PubMed PMID: 25972490; PubMed Central PMCID: PMCPMC4464746.

33. de Vries M, Van Rijckevorsel DC, Vissers KC, Wilder-Smith OH, Van Goor H. Single dose delta-9-tetrahydrocannabinol in chronic pancreatitis patients: analgesic efficacy, pharmacokinetics and tolerability. British journal of clinical pharmacology. 2016;81(3):525-37. Epub 2015/10/28. doi: 10.1111/bcp.12811. PubMed PMID: 26505163; PubMed Central PMCID: PMCPMC4767190.

34. Herrmann N, Ruthirakuhan M, Gallagher D, Verhoeff N, Kiss A, Black SE, et al. Randomized Placebo-Controlled Trial of Nabilone for Agitation in Alzheimer's Disease. The American journal of geriatric psychiatry : official journal of the American Association for Geriatric Psychiatry. 2019;27(11):1161-73. Epub 2019/06/12. doi: 10.1016/j.jagp.2019.05.002. PubMed PMID: 31182351.

35. van den Elsen GAH, Ahmed AIA, Verkes RJ, Feuth T, van der Marck MA, Olde Rikkert MGM. Tetrahydrocannabinol in Behavioral Disturbances in Dementia: A Crossover Randomized Controlled Trial. The American journal of geriatric psychiatry : official journal of the American Association for Geriatric Psychiatry. 2015;23(12):1214-24. Epub 2015/11/13. doi: 10.1016/j.jagp.2015.07.011. PubMed PMID: 26560511.

36. Peball M, Krismer F, Knaus HG, Djamshidian A, Werkmann M, Carbone F, et al. Non-Motor Symptoms in Parkinson's Disease are Reduced by Nabilone. Annals of neurology. 2020;88(4):712-22. Epub 2020/08/07. doi: 10.1002/ana.25864. PubMed PMID: 32757413; PubMed Central PMCID: PMCPMC7540547 from AOP Orphan Pharmaceuticals AG, which manufactures the drug that is tested in this study. A.D. and B.H. received a travel grant from AOP Orphan Pharmaceuticals AG, which manufactures the study drug. K.K. is an employee at AOP Orphan Pharmaceuticals AG, which manufactured the study drug. K.S. reports personal fees from AOP Orphan Pharmaceuticals AG that manufactures the drug tested in this study. F.K., H.G.K., M.W., F.C., P.E., K.M., D.V., H.S., G.G., H.U., H.K., G.K.W., R.S., and W.P. have no conflict of interest to report.

37. Carroll CB, Bain P, Teare L, Liu X, Joint C, Wroath C, et al. Cannabis for dyskinesia in Parkison disease: A randomized double-blind crossover study. Neurology. 2004;63(7):1245-50. PubMed PMID: 2004-19648-015.

38. Vaney C, Heinzel-Gutenbrunner M, Jobin P, Tschopp F, Gattlen B, Hagen U, et al. Efficacy, safety and tolerability of an orally administered cannabis extract in the treatment of spasticity in patients with multiple sclerosis: a randomized, double-blind, placebo-controlled, crossover study. Multiple sclerosis (Houndmills, Basingstoke, England). 2004;10(4):417-24. Epub 2004/08/26. doi: 10.1191/1352458504ms1048oa. PubMed PMID: 15327040.

39. Wade DT, Makela P, Robson P, House H, Bateman C. Do cannabis-based medicinal extracts have general or specific effects on symptoms in multiple sclerosis? A double-blind, randomized, placebo-controlled study on 160 patients. Multiple sclerosis (Houndmills, Basingstoke, England). 2004;10(4):434-41. Epub 2004/08/26. doi: 10.1191/1352458504ms1082oa. PubMed PMID: 15327042.

40. Blake DR, Robson P, Ho M, Jubb RW, McCabe CS. Preliminary assessment of the efficacy, tolerability and safety of a cannabis-based medicine (Sativex) in the treatment of pain caused by rheumatoid arthritis. Rheumatology (Oxford, England). 2006;45(1):50-2. Epub 2005/11/12. doi: 10.1093/rheumatology/kei183. PubMed PMID: 16282192.

41. Nurmikko TJ, Serpell MG, Hoggart B, Toomey PJ, Morlion BJ, Haines D. Sativex successfully treats neuropathic pain characterised by allodynia: a randomised, double-blind, placebo-controlled clinical trial. Pain. 2007;133(1-3):210-20. Epub 2007/11/13. doi: 10.1016/j.pain.2007.08.028. PubMed PMID: 17997224.

42. Duran M, Perez E, Abanades S, Vidal X, Saura C, Majem M, et al. Preliminary efficacy and safety of an oromucosal standardized cannabis extract in chemotherapy-induced nausea and vomiting. British journal of clinical pharmacology. 2010;70(5):656-63. Epub 2010/11/03. doi: 10.1111/j.1365-2125.2010.03743.x. PubMed PMID: 21039759; PubMed Central PMCID: PMCPMC2997305.

43. Johnson JR, Burnell-Nugent M, Lossignol D, Ganae-Motan ED, Potts R, Fallon MT. Multicenter, double-blind, randomized, placebo-controlled, parallel-group study of the efficacy, safety, and tolerability of THC:CBD extract and THC extract in patients with intractable cancer-related pain. Journal of Pain & Symptom Management. 2010;39(2):167-79. doi: 10.1016/j.jpainsymman.2009.06.008. PubMed PMID: 105129735. Language: English. Entry Date: 20100521. Revision Date: 20150711. Publication Type: Journal Article.

44. Notcutt W, Langford R, Davies P, Ratcliffe S, Potts R. A placebo-controlled, parallel-group, randomized withdrawal study of subjects with symptoms of spasticity due to multiple sclerosis who are receiving long-term Sativex(R) (nabiximols). Multiple sclerosis (Houndmills, Basingstoke, England). 2012;18(2):219-28. Epub 2011/09/01. doi: 10.1177/1352458511419700. PubMed PMID: 21878454.

45. Zajicek JP, Hobart JC, Slade A, Barnes D, Mattison PG. Multiple sclerosis and extract of cannabis: results of the MUSEC trial. Journal of neurology, neurosurgery, and psychiatry. 2012;83(11):1125-32. Epub 2012/07/14. doi: 10.1136/jnnp-2012-302468. PubMed PMID: 22791906.

46. Lynch ME, Cesar-Rittenberg P, Hohmann AG. A double-blind, placebo-controlled, crossover pilot trial with extension using an oral mucosal cannabinoid extract for treatment of chemotherapy-induced neuropathic pain. Journal of pain and symptom management. 2014;47(1):166-73. Epub 2013/06/08. doi: 10.1016/j.jpainsymman.2013.02.018. PubMed PMID: 23742737.

47. Serpell M, Ratcliffe S, Hovorka J, Schofield M, Taylor L, Lauder H, et al. A double-blind, randomized, placebo-controlled, parallel group study of THC/CBD spray in peripheral neuropathic pain treatment. European journal of pain (London, England). 2014;18(7):999-1012. Epub 2014/01/15. doi: 10.1002/j.1532-2149.2013.00445.x. PubMed PMID: 24420962.

48. Lichtman AH, Lux EA, McQuade R, Rossetti S, Sanchez R, Sun W, et al. Results of a Double-Blind, Randomized, Placebo-Controlled Study of Nabiximols Oromucosal Spray as an Adjunctive Therapy in Advanced Cancer Patients with Chronic Uncontrolled Pain. Journal of pain and symptom management. 2018;55(2):179-88.e1. doi: <https://doi.org/10.1016/j.jpainsymman.2017.09.001>.

49. Riva N, Mora G, Sorarù G, Lunetta C, Ferraro OE, Falzone Y, et al. Safety and efficacy of nabiximols on spasticity symptoms in patients with motor neuron disease (CANALS): a multicentre, double-blind, randomised, placebo-controlled, phase 2 trial. The Lancet Neurology. 2019;18(2):155-64. Epub 2018/12/18. doi: 10.1016/s1474-4422(18)30406-x. PubMed PMID: 30554828.

50. Markovà J, Essner U, Akmaz B, Marinelli M, Trompke C, Lentschat A, et al. Sativex as add-on therapy vs. further optimized first-line ANTispastics (SAVANT) in resistant multiple sclerosis spasticity: a double-blind, placebo-controlled randomised clinical trial. International Journal of Neuroscience. 2019;129(2):119-28. doi: 10.1080/00207454.2018.1481066.

51. Consroe P, Laguna J, Allender J, Snider S, Stern L, Sandyk R, et al. Controlled clinical trial of cannabidiol in Huntington's disease. Pharmacology, biochemistry, and behavior. 1991;40(3):701-8. Epub 1991/11/01. PubMed PMID: 1839644.

**Fig A in S1 Text: Funnel plotsfor all tolerability and safety outcomes: THC studies**

i. All causes Adverse Events (AEs); ii. Treatment-related AEs; iii. All cause Serious Adverse Events (AAEs); iv. Treatment-related SAEs; v. AE-related Withdrawals; vi. Deaths.

**

**Fig B in S1 Text. THC dose related withdrawals in THC studies**

**Fig C in S1 Text. Forest Plot of all cause Adverse Events: THC studies (participants with ≥50 years of age).**

Numbers under the ‘Subjects (n)’ column refer to analysed subjects from the active and control intervention arms respectively.

**Fig D in S1 Text. Forest Plot of treatment-related Serious Adverse Events: THC studies (participants with ≥50 years of age) .**

Numbers under the ‘Subjects (n)’ column refer to analysed subjects from the active and control intervention arms respectively.

**Fig E in S1 Text. Forest Plot of all cause Serious Adverse Events: THC studies (participants with ≥50 years of age).**

Numbers under the ‘Subjects (n)’ column refer to analysed subjects from the active and control intervention arms respectively.

**Fig F in S1 Text. Forest Plot of treatment-related Serious Adverse Events: THC studies (participants with ≥50 years of age).**

Numbers under the ‘Subjects (n)’ column refer to analysed subjects from the active and control intervention arms respectively.

**Fig G in S1 Text. Forest Plot of Adverse Event-related Withdrawals: THC studies (participants with ≥50 years of age).**

Numbers under the ‘Mean Age (yrs)’ and ‘Withdrawals (n)’ columns refer to the values in active and control intervention arms respectively.

The conditions listed are the disease conditions sub-grouped into broader categories for meta-regression analyses purposes. They are: Neurodegenerative disorders (ND) (dementia, Alzheimer’s disease, Parkinson’s disease (PD), Huntington’s disease, Amyotrophic lateral sclerosis); Multiple sclerosis (MS); Cancer (cancer or chemotherapy related anorexia, pain or nausea/vomiting, chemosensory alterations); and Other (type 2 diabetes mellitus, fibromyalgia, raised intraocular pressure, cervical dystonia, healthy, pancreatitis, obstructive sleep apnoea).

**Fig H in S1 Text. Forest Plot of all deaths: THC studies (participants with ≥50 years of age).**

Numbers under the ‘Subjects (n)’ column refer to analysed subjects from the active and control intervention arms respectively.

**Fig I in S1 Text. Forest Plot of all cause Adverse Events: THC:CBD studies (excluding THCV).**

Numbers under the ‘Subjects (n)’ column refer to analysed subjects from the active and control intervention arms respectively. *CE* refers to cannabis extract.

**Fig J in S1 Text. Forest Plot of treatment-related Adverse Events: THC:CBD studies (excluding THCV).**

Numbers under the ‘Subjects (n)’ column refer to analysed subjects from the active and control intervention arms respectively. *CE* refers to cannabis extract.

**Fig K in S1 Text. Forest Plot of all cause Serious Adverse Events: THC:CBD studies. (excluding THCV)**.

Numbers under the ‘Subjects (n)’ column refer to analysed subjects from the active and control intervention arms respectively. *CE* refers to cannabis extract.

 

**Fig L in S1 Text. Forest Plot of treatment-related Serious Adverse Events: THC:CBD studies (excluding THCV).**

Numbers under the ‘Subjects (n)’ column refer to analysed subjects from the active and control intervention arms respectively. *CE* refers to cannabis extract.

**Fig M in S1 Text. Forest Plot of all Withdrawals: THC:CBD studies (excluding THCV).**

Numbers under the ‘Mean Age (yrs)’ and ‘Withdrawals (n)’ columns refer to the values in active and control intervention arms respectively.

The conditions listed are the disease conditions sub-grouped for meta-regression analyses purposes are: Multiple sclerosis (MS); motor neuron disease (MND); pain (neuropathic pain, rheumatoid arthritis), cancer (cancer or chemotherapy related anorexia, pain or nausea/vomiting), diabetes mellitus, chronic obstructive pulmonary disease (COPD), healthy controls (HC), levodopa induced dyskinesia in Parkinson’s disease) (PD).

**Fig N in S1 Text. Forest Plot of all deaths: THC:CBD studies (excluding THCV).**

Numbers under the ‘Subjects (n)’ column refer to analysed subjects from the active and control intervention arms respectively. *CE* refers to cannabis extract.

**Fig O in S1 Text: Funnel plots for all tolerability and safety outcomes: THC:CBD studies**

i. All cause Adverse Events (AEs); ii. Treatment-related AEs; iii. All cause Serious Adverse Events (AAEs); iv. Treatment-related SAEs; v. AE-related Withdrawals; vi. Deaths.

**Fig P in S1 Text. THC dose related withdrawals in THC:CBD studies**

**Fig Q in S1 Text. CBD dose related all cause AEs in THC:CBD studies**

**Fig R in S1 Text. Forest Plot of all cause Adverse Events: THC:CBD studies (participants with ≥50 years of age).**

Numbers under the ‘Subjects (n)’ column refer to analysed subjects from the active and control intervention arms respectively. *CE* refers to cannabis extract.

**Fig S in S1 Text. Forest Plot of treatment-related Adverse Events: THC:CBD studies (participants with ≥50 years of age).**

Numbers under the ‘Subjects (n)’ column refer to analysed subjects from the active and control intervention arms respectively. *CE* refers to cannabis extract.

**Fig T. in S1 Text Forest Plot of all cause Serious Adverse Events: THC:CBD studies (participants with ≥50 years of age).**

Numbers under the ‘Subjects (n)’ column refer to analysed subjects from the active and control intervention arms respectively. *CE* refers to cannabis extract.

 

**Fig U in S1 Text. Forest Plot of treatment-related Serious Adverse Events: THC:CBD studies (participants with ≥50 years of age).**

Numbers under the ‘Subjects (n)’ column refer to analysed subjects from the active and control intervention arms respectively. *CE* refers to cannabis extract.

 

**Fig V in S1 Text. Forest Plot of all Withdrawals: THC:CBD studies (participants with ≥50 years of age).**

Numbers under the ‘Mean Age (yrs)’ and ‘Withdrawals (n)’ columns refer to the values in active and control intervention arms respectively.

The conditions listed are the disease conditions sub-grouped for meta-regression analyses purposes are: Multiple sclerosis (MS); motor neuron disease (MND); pain (neuropathic pain, rheumatoid arthritis), cancer (cancer or chemotherapy related anorexia, pain or nausea/vomiting), diabetes mellitus, chronic obstructive pulmonary disease (COPD), healthy controls (HC), levodopa induced dyskinesia in Parkinson’s disease) (PD).

**Fig W in S1 Text. Forest Plot of all deaths: THC:CBD studies (participants with ≥50 years of age).**

Numbers under the ‘Subjects (n)’ column refer to analysed subjects from the active and control intervention arms respectively. *CE* refers to cannabis extract.

 **Fig X in S1 Text: Funnel plot for all cause Adverse Events (AEs): CBD studies**
