# Supplementary material for: Safety and tolerability of natural and synthetic cannabinoids in adults aged over 50 years: A systematic review and meta-analysis
Source: PLoS Med. 2021 Mar 29;18(3):e1003524. doi: 10.1371/journal.pmed.1003524 (PMC8007034; doi:10.1371/journal.pmed.1003524)
Supplement: Attachment — Submitted filename: Response to reviewers 25.11.2020, final.docx [file pmed.1003524.s003.docx]

To	25.11.2020

Prof Clare Stone

Editor-in-Chief

PLOS Medicine

Dear Editor-in-Chief

Ref: Manuscript Reference Number: **PMEDICINE-D-20-04864R1**

Title: **Safety and tolerability of natural and synthetic cannabinoids in older adults: a systematic review and meta-analysis**

Thank you very much for reviewing the manuscript and for the valuable comments to further improve our paper. Please find attached the revised manuscript for re-submission.

Detailed explanations about our revisions are included in the following RESPONSE TO REVIEWERS section. We hope that you will find that our responses to comments from the editorial team and reviewers have adequately addressed the issues raised.

We look forward to hearing from you in due course.

Sincerely yours,

Sagnik Bhattacharyya

RESPONSE TO REVIEWERS:

Requests from the editors:

Editor1.1. Please update your search to a date in the past 3 months, say.

Response: We have updated the search until 31^st^ Oct 2020 which yielded 3 additional studies. We have reanalysed and found that the results are essentially similar. We have updated our results, tables and figures accordingly.

Editor1.2. Please convert your abstract into the three-part PLOS Medicine style. The final sentence of the "methods and findings" subsection should quote 2-3 of the study's main limitations.

Response: We have now amended this.

Editor1.3. Are you able to quote aggregate participant demographic details in the abstract? Please add brief information about where the trials were done.
Response: We have now added this information in the abstract:

“46 RCTs were identified as suitable for inclusion of which 31 (67%) were conducted in the UK and Europe. There were 6216 patients (mean age 58.6 years; 51% male) included in the analysis, with 3469 receiving CBMs.”

Editor1.4. Please begin the final subsection with "In this study, we found that ..." or similar.

Response: We have amended the subsection as suggested, starting with: “This pooled analysis suggests that although…”

Editor1.5. After the abstract, we ask you to add a new and accessible "author summary" section in non-identical prose. You may find it helpful to consult one or two recent research papers in PLOS Medicine to get a sense of the preferred style.

Response: We have now added this section.

Editor1.6. Please note in your methods section that ethics approval was not required for this analysis (assuming that this is the case).

Response: We have added a sentence at the end of first para in methods section, “Ethics approval was not required for this systematic analysis”.

Editor1.7. Please restructure the paragraph on limitations in the discussion section so as to discuss limitations of the present analysis in addition to those of the studies included.

Response: We have now addressed this and added limitations of the present analysis under Limitations and Strengths subsection of the Discussion section,

“Our review and meta-analysis are limited by a number of methodological weaknesses some of which stem from the design and analytic approach of the present study and others which are related to weaknesses in the studies that were included in the present analysis.

One of the key limitations inherent in the meta-analytic approach that also applies to the present study relates to the issue of heterogeneity in the pooled data [95]. Studies pooled in our analyses focused on patients with different clinical conditions and indications, used different doses, formulations and routes of administration the study drug for different treatment periods and employed different study designs (cross-over versus parallel-arm RCTs). Further, although we attempted to control for patients from widely disparate age groups taking part in the trial by setting an inclusion criterion of mean age of 50 years and over as the cut-off, studies included in our main analyses still involved data from people below age 50 and studies also varied in terms of gender distribution. This is reflected in the heterogeneity estimates reported in our results. In order to address this wide-ranging heterogeneity and yet pool the data in a meaningful way, we employed a random-effects model in our analyses, which assumes that there will be variability in the observed estimates of treatment effects across studies, both as a result of real differences in the effect of treatment between studies as well as by chance because of sampling variability. As a result, pooled estimates reported are not precise (as may be evident from the wide confidence intervals) and should not be considered as such. Effects reported here represent an average effect of CBM treatments on safety and tolerability outcomes investigated rather than an effect that is common across studies. As the effects may be different within an individual study, we also report a prediction interval for our key reported outcomes, in order to give an estimate of the range of predicted effects across study settings that may be observed in a new study. Further, we also carried out sub-group (e.g. cross-over and parallel-arm RCTs separately) and meta-regression analyses to examine the sources of heterogeneity for THC and THC:CBD studies. These analyses did not suggest that study design or type of intervention had any significant effect on the outcomes assessed, though they suggest that effects vary, sometimes significantly, across clinical conditions investigated in the RCTs. They were more pronounced in AE-related withdrawals and treatment-related AEs in THC:CBD studies and less so for all cause AEs in studies investigating THC as a treatment. Dose of study drug also seemed to underlie some of the heterogeneity observed in all cause and treatment-related AEs and AE-related withdrawals in the THC and THC:CBD studies. Another limitation relates to the fact that we were not able to systematically examine the sources of heterogeneity for CBD alone studies as there were fewer studies than recommended for such analyses. One other limitation of the present meta-analysis relates to our focus on studies reporting on participants aged 50 years and over.

We included studies in which the mean age of study participants was ≥50 years (although the studies also included many participants who were <50 years of age). As the cut-off employed by us differs from the conventional threshold of 65 years for ‘elderly’ [96] may be considered as a limitation. However, this was chosen as the clinical conditions (diabetes, cancer, neurodegenerative disorders, cancer etc) for which CBMs are often considered become more common from around this age. This is also a period characterised by multi-morbidities, polypharmacy and age-related bodily changes that may affect pharmacokinetics [96] and tolerability of medications. Further, in order to address the limitation that many participants in the included studies were <50 years of age, we also carried out sensitivity analysis that included studies where all participants were ≥ 50 years of age. The results of these analyses suggest that the pooled effect-sizes were generally in the same direction as that reported from the larger set of studies, though the confidence intervals were wider, as may be expected. We have also reported the number of studies which have actually studied individuals with age ≥ 65 years or ≥ 75 years. As evident from this, there is a very limited set of studies that have exclusively focused on people at these ages. Therefore, the present meta-analysis highlights the need for studies that may need to focus on people over 65. However, given the age range as well as median and interquartile range of the mean ages of study participants included in the studies that constitute our meta-analysis, it is clear that people over 65 and 75 years are currently being recruited into studies of CBMs for various indications. As individual RCTs are often not powerful enough to unravel patterns of side-effects and given the growing use of CBMs in the elderly and general perception of them being safe, it is particularly important to summarize currently available evidence to help inform about the safety and tolerability profile of CBMs in those aged 50 years and over rather than wait for the evidence base focusing only on ≥ 65 years to mature. In the fullness of time, future attempts at evidence synthesis need to focus only on studies of people ≥ 65 years when a sufficient number of studies have accumulated.”

Editor1.8. Please remove the information on author contributions, funding and competing interests from the end of the main text. In the event of publication, this information will appear in the article meta-data via entries in the submission form.

Response: We have removed them.

Editor1.9. Throughout the text, please convert reference call-outs to the following style: "... younger people [9,10]." (i.e., the square brackets should be preceded by a space but contain no spaces).

Response: Done.

Editor1.10. In your reference list, please abbreviate journal names consistently (e.g., "PLoS Med."; and "Lancet" will suffice for reference 78).

Response: Done

Editor1.11.Please adapt the attached PRISMA checklist so that individual items are referred to by section (e.g., "Methods") and paragraph number rather than by line or page numbers, as the latter generally change in the event of publication. Please refer to the checklist early in the methods section of your main text ("See S1_PRISMA_Checklist" or similar).

Response: Done.

Comments from Academic Editor:
Editor 2.1. Page 4: The authors write "THC is addictive(4)...". Please use a more specific word or set of words in lieu of the word "addictive" -- are the authors referring to physiological dependence? Psychological dependence? Both? Compulsive use despite negative consequences? Something else?

Response 1: In response to this comment and comment from reviewer 3 (below), we have now amended the term in the manuscript and referenced it (2^nd^ paragraph under Introduction section), “THC can cause intoxication [4, 5] and has anti-emetic, analgesic and potentially neuroprotective and anti-inflammatory effects.”

Editor 2.2. Page 4: Same as above with the statement "CBD is non-addictive(5)..."
Response 2: We have amended this too as , “On the other hand, CBD is non- intoxicating [5, 6] with antiepileptic…..”

Editor 2.3. Page 6: As with R2, I have questions about the focus of this review to studies of "older adults", defined as studies with "mean age >=50 years". This inclusion criterion is based on an individual-level variable, ie, the authors did not restrict their meta-analysis to studies in which the age cutoff for participation was age >=50 years. Thus, many of the studies included in this meta-analysis involve much younger individuals. For example, the study by Curtis et al. 2009 had a mean age of 52 years (SD, 9.5 years), thus assuming the distribution of ages was normal we would expect approximately 16% of participants were <42 years of age. The study by Lane et al. 1991 appears to include study participants with an age range of 22-64 years. There are other examples. The authors seem to be aware of this distinction, given that in the Supplementary Appendix they write (for example): "These results are also consistent with a prospective observational study of 901 people above 65
years of age (74.5 ± 7.5 years)..."-- in that sentence it is clear that the study restricted enrollment to people >65 years of age and that the mean age of study participants was also >65 years. Yet on Page 14 the authors cite their specific focus on older-age people to distinguish their meta-analysis from two previously published meta-analyses (refs #15 and #16). The JAMA paper does not AFAIK report mean ages, but in the CMAJ paper, the mean ages for most of the included studies appear to be roughly in the same range as those included in the current meta-analysis.

To fix this, I think the authors need to do the following:

Editor 2.3a) I think the authors should consider restricting this meta-analysis to studies where age >=50 years was one of the inclusion criteria.

Response 2.3a: Thank you for allowing us to address this. In response to this comment as well as from reviewer 2 below, we have now also reported the results of analyses restricted to studies where all participants were clearly aged 50 years and over as well as from studies with participants aged ≥ 65 years in addition to our originally reported results. There is a larger evidence base of studies with mean age of participants ≥ 50 years than of studies where all participants were ≥ 50 years of age (and even fewer for ≥ 65 years). Therefore, we feel that reporting both sets of results would be more informative than focusing on the smaller set of studies (where all participants were ≥ 50 years of age) for which the evidence base is yet to mature. Our results suggest that the pooled effects estimated from studies where all participants were ≥ 50 years (or ≥ 65 years) of age were broadly comparable in terms of direction of effects with the results derived from the larger meta-analytic set of studies where only the mean age of study participants was ≥ 50 years. There were some differences as well, which we have highlighted and discussed. As evident from these analyses, there is a more limited set of studies that have exclusively focused on people over 50 years and even less on people over 65 or 75 years. Therefore, the present meta-analysis highlights the need for studies that may need to exclusively focus on older people and in particular people over 65. However, given the age range as well as median and interquartile range of the mean ages of study participants included in the studies that constitute our meta-analysis, it is clear that people over 65 and 75 years are currently being recruited into studies of CBMs for various indications. As individual RCTs are often not powerful enough to unravel patterns of side-effects and given the growing use of CBMs in the elderly and general perception of them being safe, it is particularly important to examine this by summarizing currently available evidence to help inform about the safety and tolerability profile of CBMs in those aged 50 years and over rather than wait for the evidence base to mature. In the fullness of time, future attempts at evidence synthesis may need to focus only on studies of people ≥ 65 years when a sufficient number of studies have accumulated. Therefore, we have taken the approach to report our results as originally reported (however with an updated search including additional studies) but have also reported the pooled estimates from studies exclusively focusing on people aged ≥ 50 years as well as aged ≥ 65 years.

We have also elaborated on the rationale for presenting the results as above in the text and amended the Introduction, Methods and Discussion sections accordingly.

Under Introduction, 3^rd^ paragraph:

“…..that focused on people with mean age of 50 years and over to conduct a meta-analysis. As there is a larger evidence base of studies with mean age of participants ≥ 50 years than the more limited set of studies that have exclusively focused on people over 50 years and even less on people over 65 or 75 years, we have focused on studies with mean age of participants ≥ 50 years and complemented these results with additional analyses restricted to studies that have exclusively focused on people over 50 years and even less on people over 65. …”

Under Methods, Data synthesis and analysis sub-section, 3rd paragraph:

“Our primary analysis includes the results of all studies where the mean age of study participants was ≥50 years. As many participants in these included studies were <50 years of age, we also carried out separate analyses restricted to studies where all participants were ≥ 50 years of age and also where all participants were ≥ 65 years of age. These analyses were carried out where there were at least 2 studies with analysable data…..”

Please also see responses to comment Editor 2.3d, for amendments and additions in Results and Discussion sections

If the authors decide not to do the above, and provide reasonable justification for not doing so, then I think the authors need to do the following:

Editor 2. 3b) Make it clear in the Methods and Discussion that their inclusion criterion was *mean* age >=50 years. There are places in the manuscript where this is not always clear. For example, on page 15, the authors write: "Our use of a lower cut-off age of 50 years to define the target population..." This sentence somewhat obscures the limitation here. A more precisely worded sentence would be something like "We included studies in which the mean age of study participants was >=50 years (although the studies included many participants who were <50 years of age), and this cutoff differs from the conventional threshold of 65 years..."

Response 2.3b: Thank you for the comment. We have amended the section containing this sentence, which now reads as: “Further, we included studies in which the mean age of study participants was ≥50 years (although the studies also included many participants who were <50 years of age). As the cut-off employed by us differs from the conventional threshold of 65 years for ‘elderly’ [96], this….”

Editor 2.3c) The tables should split the rows into two categories: "Trials in which enrollment was restricted to age >=50 years" and "Trials in which enrollment was not restricted to age >=50 years, but the mean age of study participants was >=50 years".

Response 2.3c: We have amended Appendix Table 2 a and b and split the rows into the two categories as suggested. We have added column ‘Age stratification’, with rows for ‘Mean age ≥50 years’ and ‘Age ≥50 years’.

In addition, to the Tables 1 a-c in the manuscript we have added asterisk to the studies to indicate- **Studies recruited participants ≥50years; ***Studies recruited participants ≥65 years.

Editor 2.3d) If adequately powered, subgroup meta-analyses for the two categories above should be conducted (and reported in the main text if substantively different from the primary analysis; or perhaps reported in an Appendix if the pattern of findings is largely similar to the primary analysis). If subgroup analyses are not possible, the Results text should at least identify the number of RCTs and patients in which enrollment was restricted to age >=50 years.

Response 2.3d: There were 4 THC RCTS which recruited participants over ≥50 years,

(one ≥ 50 years; two ≥65 years, one ≥75 years) and 3 THC:CBD RCTs ( two ≥50 years; one ≥65 years). We carried out subgroup meta-analysis and found no difference to the primary analysis.

This has been included as the 6^th^ and 7^th^ paragraph under THC studies in the Results section,

“***Analysis of studies where all participants were ≥50 years:***

Restricting the meta-analysis to the four studies [39-41, 65] that recruited participants with ≥50 years of age (n=126), results were broadly comparable to the primary analysis in the pattern of findings, though effect on all cause AEs was no longer significant. The pooled IRRs for all cause AEs (k=3) was 2.13 (95% CI, 0.46 -9.97) and treatment-related AEs (k=3) was 2.80 (95% CI, 1.09 -7.21) respectively. Pooled IRRs for all cause (k=4) and treatment related SAEs (k=2) were 1.20 (95% CI, 0.39 -3.65) and 0.50 (95% CI, 0.03 -7.99) respectively. Pooled RR for AE-related withdrawals (k=4) and IRR for all deaths (k=4) were 1.11 (95% CI, 0.49 -2.55) and 0.58 (95% CI, 0.11 -3.06) respectively (eFigure 9 a-f).

***Analysis of studies where all participants were ≥65 years:***

Restricting the meta-analysis to only the three studies [39-41] that recruited participants with ≥65 years of age (n=126), effects were also broadly comparable to the primary analysis in the pattern of findings, with the exception of effect on all cause AEs, which was no longer significant. The pooled IRRs for all cause AEs (k=3) was 2.13 (95% CI, 0.46 -9.97) and treatment-related AEs (k=2) was 2.80 (95% CI, 1.09 -7.21) respectively. Pooled IRRs for all cause (k=3) and treatment related SAEs (k=2) were 1.80 (95% CI, 0.13 -8.76) and 0.50 (95% CI, 0.03 -7.99) respectively. Pooled RR for AE-related withdrawals (k=3) and IRR for all deaths (k=3) were 1.00 (95% CI, 0.23 -4.41) and 0.42 (95% CI, 0.05 -3.42) respectively.”

And also included as the 6^th^ and 7^th^ paragraphs under THC:CBD combination studies in the Results section,

“***Analysis of studies where all participants were ≥50 years:***

Restricting the meta-analysis to the 3 studies [42, 68] that recruited participants with ≥50 years of age (total n= 60; analysed n=52) were similar to the primary analysis in the pattern of findings, except for treatment-related AEs and AE-related withdrawals, which were no longer significant. The pooled IRRs for all cause AEs (k=3) was 2.65 (95% CI,1.49 -4.71) and treatment-related AEs (k=2) was 4.73 (95% CI, 0.54 -41.65) respectively. Pooled IRRs for all cause (k=3) and treatment related SAEs (k=3) were 1.00 (95% CI, 0.10 -9.61) and 1.00 (95% CI, 0.10 -9.61) respectively. Pooled RR for AE-related withdrawals (k=3) and IRR for all deaths (k=3) were 0.77 (95% CI, 0.18 -3.22) and 1.00 (95% CI, 0.10 -9.61) respectively (supplement eFigure 10 a-f). None of these studies reported any SAEs (either treatment-related or all cause) or death.

***Analysis of studies where all participants were ≥65 years:***

“As analysable data was available from only one study, this analysis was not carried out.”

Under CBD studies in the Result section,

“No analysable data was available from studies where all study participants were ≥50 years age.”

Under Discussion section, 2^nd^ paragraph:

“Additional analyses restricted to only those studies in which all participants were ≥50 years of age or ≥ 65 years of age, where this was feasible, indicated a pattern of findings broadly comparable with the results of our main analyses including all studies. However, effect of CBMs containing THC but no CBD on all cause AEs and CBMs containing THC:CBD on treatment-related AEs and AE-related withdrawals were no longer significant, which likely reflects the lower power of these analyses which included a maximum of 4 studies in any analysis.”

Under Discussion section, last sentence in 3^rd^ paragraph:

“It is important to note that although the general pattern of results of analyses restricted to studies where all participants were ≥50 years of age or ≥ 65 years of age were broadly comparable with results from our main analyses of studies with mean participant age ≥50 years, they were not identical, indicating that effects of CBMs may be different when focused exclusively on adults over 65 or 75 years of age.”

Editor 2.3e) A column should be added to the tables showing the age cutoff for enrollment (eg., >18 years, >40 years, "none", etc)

Response 2.3e: Thank you for the suggested. We have added a column to Table 1 a, b and c with a column for “Age cut-off for enrolment”.

Editor 2.3f) As suggested by R2, the Results text should identify the number of RCTs and patients in which enrollment was restricted to age >=65 years, and/or >=75 years. (If there were no trials in which
Response 2.3f: Thank you. This comment seems to have got truncated abruptly. However, we have now identified these in the text of the Results section: (1^st^ paragraph, last sentence), “Of these, 4 RCTs recruited participants over age ≥ 65 years (n=34; mean age, 72.4 (SD± 4.5)), of which one was ≥ 75 years (Table 1a and b) [39-42]”.

Editor 2.3g) The same modifications to the tables should be done for the Appendix Table 1. For example Appendix Tables 2A and 2B should report the N (%) of studies that were restricted to age >=50 years and age >=65 years.

Response 2.3g: Appendix table 1 summarizes the unpublished trials with information obtained from clinicaltrials.gov website and therefore limited information is available and not included in the meta-analysis. The mean age for all these trials was ≥50 (we have now added a column for mean age), although the age cut-off for recruitment was ≥18 years.

Appendix table 2; We have indicated the N(%) in the title and also included additional rows for this group as suggested in comment 2.3c.

Editor 2.4. Page 8: The authors report that the mean age of treatment-arm study participants was "mean± SD: 44.39±190.52 person-years". How did they calculate the pooled mean age here? This detail is not reported in the Methods. They report pooled mean ages later on in the manuscript as well, eg page 11, Appendix Table 2A, etc.

Response 2.4: Thank you. We trust that the academic editor is referring to this section (shown in BOLD) in the previous version: “A total of 29 RCTs (15 crossover and 14 parallel-arm) from 28 articles(31-57) (see Supplemental Results and Supplemental Table 2a, for additional details), reported on 1442 patients on active (analysed 1390; 1243.03 person-years; **mean± SD: 44.39±190.52 person-years**) and 1232 (analysed 1182) patients on control intervention,…”

Apologies for the confusion. We have reported the total person-years of exposure to the active intervention (THC in this case) and the not the mean age. In response to this comment, we have now clarified that the numbers reported in this context refer to total and mean exposure to active intervention in person-years within the Results section for THC, THC:CBD and CBD studies, 1^st^ paragraph each. (it is worth noting that the actual numbers have changed with the new studies following an updated search).

Pooled mean ages of participants for each group of studies (and treatment arms) that have been reported throughout the manuscript have been estimated by calculating the arithmetic mean of study specific mean age as reported by individual studies (as reported e.g. in Tables 1a, 1b & 1c) for each reported group and intervention arm.

As suggested, we have amended the Methods section to describe how these were calculated, under Data Synthesis and Analysis sub-section, 1^st^ paragraph,

“Total exposure to active intervention in person-years was estimated by first calculating this for each individual study by multiplying the number of subjects in the active intervention arm with the duration of treatment for that arm for each study and then adding up these study-specific values for all studies under each broad category (THC, THC:CBD or CBD) of intervention investigated here. Mean exposure in person-years for each category (THC, THC:CBD or CBD) of intervention was estimated by calculating the arithmetic mean from study-specific estimates obtained as above for each intervention category. Pooled mean ages of participants for each group of studies and treatment arms were estimated by calculating the arithmetic mean of study specific mean age as reported by individual studies for each intervention arm. Other pooled estimates (median and interquartile range) for summary study characteristics , such as, duration of study in weeks, participants analysed or included, or duration of treatment for each treatment arm (as reported in Supplementary Material; Table 2A), were calculated by estimating them from the total number (e.g. for variables such as number of participants included or analysed; duration of study or treatment) or the mean estimate (e.g. for variables such as mean age) as reported in individual studies for each set sub groups of RCTs.“

Editor 2.5. On a related note, the authors report other pooled mean estimates (eg., Appendix Table 2A, pooled mean duration of study in weeks)-- how are these estimated?

Response 2.5: We have now addressed this as we have stated in the response to comment 2.4 above.

Editor 2.6. Figures 2A-2G: Figure 2A reports very high levels of heterogeneity (I2>73%), but Figures 2B-2F report no heterogeneity at all (I2=0%). Is this a typo? The patterning of the figures does not seem consistent with I2's of 0%.

Response 2.6: Thank you. We have checked the analysis results and the code and the I2 estimates that read as ‘0%’ were as obtained in our analyses. As the I2 estimate is equal to ((Q-degree of freedom)/ Q) expressed as a percentage (where Q refers to Cochran’s Q, the heterogeneity test statistic), and as negative values of I2 are set as equal to zero (Higgins et al., BMJ 2003), such that I2 values lie between 0 and 100%, we believe that the I2 estimate has been 0% in those analyses where the Q statistic value has been smaller than the degrees of freedom. We have also reported the Q statistic, degrees of freedom and p values of the heterogeneity test in the forest plots to inform the reader. As would be evident, these did not suggest significant heterogeneity as per the significance of Q statistic for the relevant analyses. We appreciate the editor’s point that I2 estimates are not consistent with the pattern of certain results shown in forest plots. This is likely because none of these estimates are perfect and have their limitations (Higgins et al 203, von Hippel , 2015). Therefore, we have also reported the prediction intervals for even those studies where I2 estimates read as 0% and not just the analyses with high I2 estimates where this was advised (please see our response to comment 2.6 below).

Editor 2.6a) Given the high levels of heterogeneity (reported in Figure 2A at least), I would suggest including prediction intervals:
https://eur03.safelinks.protection.outlook.com/?url=https%3A%2F%2Flink.springer.com%2Farticle%2F10.1007%2Fs10654-012-9738-y&amp;data=04%7C01%7Csagnik.2.bhattacharyya%40kcl.ac.uk%7Ce62925bc2e0442141e1c08d87cf6ae8c%7C8370cf1416f34c16b83c724071654356%7C0%7C0%7C637396746216825407%7CUnknown%7CTWFpbGZsb3d8eyJWIjoiMC4wLjAwMDAiLCJQIjoiV2luMzIiLCJBTiI6Ik1haWwiLCJXVCI6Mn0%3D%7C1000&amp;sdata=kWaOlIStYb8%2BEH%2BMTgL%2FcR59gxTCNxiwfQOqpMQclWs%3D&amp;reserved=0
Response 2.6a: Thank you. We have now added prediction intervals for summary estimates in all of our forest plots (Figure 2 A-F; Figure 3 A-F; Figure 4A)

We have also referred to these in the Discussion in the 6^th^ paragraph, 2^nd^ sentence,

“…..Therefore, we have also reported the prediction intervals along with the 95% confidence intervals in the forest plots for the key pooled estimates reported herein. These prediction intervals indicate that while the average effects of CBM treatments on these outcomes are significant (as evident from the 95% confidence intervals), the range of predicted effects across different study settings that may be observed in a new study may span a wider range of effect sizes than indicated by the 95% confidence intervals. Some of these predicted effect-sizes in new studies are likely to be <1 indicating that the risk of these outcomes with THC or THC:CBD treatment may not be higher than under control treatment condition.”

Editor 2.6b) The same modifications should be done for Appendix Figure 1A & Appendix Figures 8A-8F.

Response 2.6b: Thank you. We have now added prediction intervals for summary estimates in all of our forest plots in the supplement material (Figure 7a-f; Figure 8a-f and Figure 9 a-f in S1 Appendix).

Editor 2.6c) I think the authors should also include a more detailed discussion of the heterogeneity, which is only mentioned in passing on page 15. Some would argue that such a high degree of heterogeneity would suggest _not_ pooling the data using meta-analysis:
https://eur03.safelinks.protection.outlook.com/?url=https%3A%2F%2Fjamanetwork.com%2Fjournals%2Fjamanetworkopen%2Ffullarticle%2F2758470&amp;data=04%7C01%7Csagnik.2.bhattacharyya%40kcl.ac.uk%7Ce62925bc2e0442141e1c08d87cf6ae8c%7C8370cf1416f34c16b83c724071654356%7C0%7C0%7C637396746216825407%7CUnknown%7CTWFpbGZsb3d8eyJWIjoiMC4wLjAwMDAiLCJQIjoiV2luMzIiLCJBTiI6Ik1haWwiLCJXVCI6Mn0%3D%7C1000&amp;sdata=%2FjZjnkmFbCc7LeoyL7AAPN9fLP6o1H5p5YjaKRh4XSw%3D&amp;reserved=0
<https://eur03.safelinks.protection.outlook.com/?url=https%3A%2F%2Fjamanetwork.com%2Fjournals%2Fjamanetworkopen%2Ffullarticle%2F2758468&amp;data=04%7C01%7Csagnik.2.bhattacharyya%40kcl.ac.uk%7Ce62925bc2e0442141e1c08d87cf6ae8c%7C8370cf1416f34c16b83c724071654356%7C0%7C0%7C637396746216825407%7CUnknown%7CTWFpbGZsb3d8eyJWIjoiMC4wLjAwMDAiLCJQIjoiV2luMzIiLCJBTiI6Ik1haWwiLCJXVCI6Mn0%3D%7C1000&amp;sdata=syNoN1KxfWcXOACy1mrWg6YTXRtmj09QOknw7tDso1c%3D&amp;reserved=0>

Response 2.6c: Thank you for this comment. We have now added the below as 4^th^ paragraph under Discussion in the 6^th^ paragraph,

“Pooled estimates of all cause AEs under both THC and THC:CBD and of treatment-related AEs and AE-related withdrawals under THC:CBD as reported herein need to be considered carefully in light of high-levels of heterogeneity in the studies as evident from the estimated *I^2^* statistic (>32% in all these estimates). Therefore, we have also reported the prediction intervals along with the 95% confidence intervals in the forest plots for the key pooled estimates reported herein….”

We have also added first 2 paragraphs under sub-section Limitations and Strengths, under Discussion section to address this (Please see response to comment Editor 1.7 for the text)

Editor 2.7. Figures 2G & 3G: The studies are only identified with numbers, but on first glance it is not clear what these numbers are referring to. These numbers are not listed in the other tables, obscuring the studies. I don't see the studies identified until I scrutinize the footnotes to Figures 2 & 3.

Response 2.7: Thank you for the comment. We have amended the figures as suggested in the below comments.

Editor 2.7a) The row headers for Figures 2G & 3G should instead identify the study the same way they are identifed in the other figures (eg, "Notcutt et al., 2012"). If the authors are worried about horizontal space, I would suggest formatting the figure in landscape mode.

Response 2.7a: This is amended, and the studies are identified on the figures 2G and 3G.

Editor 2.7b) In addition, the column headers for Figures 2G & 3G would benefit by simply stating the domain names (eg, "random sequence generation") instead of the abbreviation (eg., "D1"). Yes, the footnote does connect "D1" to "random sequence generation" but it is an additional cognitive step for readers. If the authors are worried about horizontal space in the column headers, they could orient the text at 90 degrees or 45 degrees.

Response 2.7b: This is now amended as suggested.

Editor 2.7c) The same modifications should be done for Appendix Figure 1B.

Response 2.7c: Done.

Editor 2.8. Appendix Table 3B & Table 4: The authors report a pooled estimate for the "immune system disorder" subgroup but the k=1-- is this a typo?

Response 2.8: Apologies for this oversight. We have now amended these.

Comments from the reviewers:

*** Reviewer #1:
I confine my remarks to statistical aspects of this paper. These were generally fine.  I have one request and some comments on the figures.

Reviewer 1.1: First, please give an effect size whereever you give a p value (e.g. you don't do this in a couple spots on p. 11).

Response1.1: Thank you. We have now added these.

Comment 1.2. For the forest plots, I would combine the active and control colums (e.g 21/21) and then abbreviate the drug (D M, T, etc( to save space. Then expand teh actual graph portion.  I also wonder how the numbers at teh bottom (0.02, 0.14 ...) were chosen rather than something more standard like 0.01, 0.05 etc.

Response 1.2: Thank you. We have combined the active and control columns and expanded the actual graph portion in the forest plots as suggested. As combining the active and control columns helped free up space for the plot such that it was no longer encroaching on to texts, we did not abbreviate the drug names (except, ‘CE’ for cannabis extract). This was also to avoid have the reader having to navigate additional abbreviations that were not immediately obvious.

But, generally, very well done

General response: Thank you for the positive comment.

Peter Flom

*** Reviewer #2:
This is an excellent systematic review and meta-analysis on the safety and tolerability of natural and synthetic cannabinoids in older adults.

General response: Thank you very much for the positive comment.

Minor comments:

Comment 2.1: Since life expectancy have increased considerably over the past and people in the age of 50 years are nowadays commonly rather fit, the reader wonders why this age was chosen for the identification of older adults? The authors briefly address this in their discussion, however, one wonders whether other factors might have determined their choice of this age (≥ 50 years), e.g. that they would not have collected enough RCTs for this meta-analysis. Thus, they could give a little more transparency whether they would have recruited enough RCTs and patients if the cut-off in age ≥ 65 years would have been chosen.
For this reason, they should clearly state the age ≥ 50 years in their final conclusions (in the abstract and end of discussion) and state that results of this meta-analysis might be different in the age group of ≥ 65 years or even ≥ 75 years.

Response 2.1: Thank you for allowing us to address this. In response to this comment as well as from the academic editor (please also see our response to comment Editor 2.3a and Editor 2.3d) , we have now also reported the results of analyses restricted to studies where all participants were clearly aged 50 years and over as well as from studies with participants aged ≥ 65 years in addition to our originally reported results. There is a larger evidence base of studies with mean age of participants ≥ 50 years than of studies where all participants were ≥ 50 years of age (and even fewer for ≥ 65 years). Therefore, we feel that reporting both sets of results would be more informative than focusing on the smaller set of studies (where all participants were ≥ 50 years of age) for which the evidence base is yet to mature. Our results suggest that the pooled effects estimated from studies where all participants were ≥ 50 years (or ≥ 65 years) of age were broadly comparable in terms of direction of effects with the results derived from the larger meta-analytic set of studies where only the mean age of study participants was ≥ 50 years. There were some differences as well, which we have highlighted and discussed. As evident from these analyses, there is a very limited set of studies that have exclusively focused on people over 50 years and even less on people over 65 or 75 years. Therefore, the present meta-analysis highlights the need for studies that may need to exclusively focus on older people and in particular people over 65. However, given the age range as well as median and interquartile range of the mean ages of study participants included in the studies that constitute our meta-analysis, it is clear that people over 65 and 75 years are currently being recruited into studies of CBMs for various indications. As individual RCTs are often not powerful enough to unravel patterns of side-effects and given the growing use of CBMs in the elderly and general perception of them being safe, it is particularly important to examine this by summarizing currently available evidence to help inform about the safety and tolerability profile of CBMs in those aged 50 years and over rather than wait for the evidence base to mature. In the fullness of time, future attempts at evidence synthesis need to focus only on studies of people ≥ 65 years when a sufficient number of studies have accumulated. Therefore, we have taken the approach to report our results as originally reported (however with an updated search including additional studies) but have also reported the pooled estimates from studies exclusively focusing on people aged ≥ 50 years as well as aged ≥ 65 years.

We have also made the changes to our abstract and discussion as suggested and indicated the number of studies in participants ≥ 65 years and ≥ 75 years.

In the Abstract:

**“**Conclusions: This pooled analysis, using data from RCTs with mean participant age ≥ 50 years, suggests that although THC-containing CBMs are associated with side-effects, CBMs in general are safe and acceptable in older adults. However, THC:CBD combinations may be less acceptable in the dose ranges used and their tolerability may be different in adults over 65 or 75 years of age.”

In the Discussion:

“Conclusion: Results of the present study using data from RCTs with mean participant age ≥ 50 years suggest that although THC-containing CBMs are associated with side-effects in those aged 50 years and over, in general CBMs are safe and acceptable treatments in older adults, with a caveat that THC:CBD combinations may be less so at least in dose ranges used in studies thus far. However, tolerability may be different in adults over 65 or 75 years of age, and robust evidence of efficacy of different CBMs for specific indications is needed before they may be used in routine practice in older adults.”

Comment 2.2: It is somewhat mysterious that the THC:CBD combination but not THC or CBD alone significantly increased the risk of AE-related withdrawals and the authors do not give a good explanation. Might this result be out of pure chance? The authors should give some explanation for this phenomenon?

Response 2.2: We have now addressed this in the Discussion section, 3^rd^ paragraph:

“... Greater risk of withdrawal from study in those receiving CBMs containing THC:CBD combination but not in those containing THC or CBD without the other cannabinoid is an unexpected finding. While we did not compare these two effects in a systematic manner, if true, this may suggest that combination of these cannabinoids may be less acceptable in this age group, who already may be receiving multiple treatments for comorbidities. What may underlie these effects is much less clear. One may speculate that this may be a result of the generally higher THC dose employed in THC:CBD studies compared to the THC studies, which may have made participants in the former group of studies more susceptible to AEs and withdrawal as a result compared to those receiving THC. This is supported by approximately double the median dose of THC used in THC:CBD studies [median and interquartile range: 10.3(10.2- 21.6) mg/ day in crossover and 20.8(14.6-25) mg/ day in parallel-arm RCTs] as opposed to THC studies [median and interquartile range: 5(2.25-9 mg/ day in crossover and 10(4.8-25) mg/ day in parallel-arm RCTs]. In accordance with this hypothesis, there was a nominally higher incidence of AEs in general in those studies using both cannabinoids in combination compared to THC studies. However, one cannot completely rule out the possibility that greater risk of withdrawal from study in those receiving CBMs containing THC:CBD combination but not in those containing THC or CBD without the other cannabinoid, as observed here, is purely by chance. Further, THC dose was significantly associated with withdrawal in both study groups using THC-containing medications. In contrast, CBD dose was only associated with AEs but not withdrawal in those using THC:CBD combination. However, whether these results suggest that lower acceptability of THC:CBD combination is a tolerability issue in older patients because of combination of cannabinoids rather than being related to the dose of CBD, remains to be formally tested. It is also worth noting that the evidence base of studies that used CBMs without THC in older adults and found it to be well-tolerated is relatively sparse. Whether this may underlie the absence of significantly greater risk of all cause AEs as a result of treatment with CBMs without THC compared to controls, contrary to our predictions, remains to be tested. However, more generally, lack of significant AEs associated with CBD treatment alone in the present study is consistent with other analyses suggesting a good tolerability profile of CBD in younger age groups [87, 88].

Comment 2.3: In their supplemental discussion the authors state "Firstly, Whiting reported odds ratios and Wang et al reported relative risk, wherein we have reported IRR which takes into consideration person-years of treatment and hence is more meaningful." The authors should address this more thoroughly, explain the differences and clinical consequences in the estimated risk to the reader in the discussion of the manuscript and not only in the

supplemental material.

Response 2.3: We have now moved the supplemental discussion to the main manuscript and have added further clarification in the 11^th^ paragraph under Discussion section:

“It is difficult to compare our pooled effect-size with previous reviews due to a number of reasons. Firstly, Whiting reported odds ratios and Wang et al reported relative risk, wherein we have reported IRR. In the present context, the risk of AEs or SAEs in either study arm are unlikely to be constant over time. Therefore, IRR which takes into consideration person-years of treatment and is a ratio of the incidence rate in the experimental treatment group to that in the control treatment group, is more meaningful and appropriate in contrast to the odds ratio, which is a ratio of the odds of an event in the experimental group to that in the control group. Secondly, we carried out a pooled analysis for individual interventions unlike the other reviews, with Wang et al also excluding studies using Nabilone[17]. Finally, the 2 previous reviews that reported summary estimates included all age groups and were not specific for older people unlike here.”

*** Reviewer #3:

Thank you for the opportunity to review this manuscript. The authors make a sound case for the need to determine the safety and tolerability of CBMs among older populations. My read of the review process and analysis seems appropriate, however I would encourage having a statistical expert in meta-analysis techniques to also review this paper. My comments are primarily around the positioning of the review, and how the results are contextualised.

General response: Thank you very much for the positive comment.

Comment 3.1: On page 5, there is reference made to growing evidence that THC and CBD may have 'opposing effects on certain organ systems' - this should be elaborated on, both in terms of outlining what this means, and what its consequences are for patients who may use different forms of CBMs. It is also noted on page 4 that 'THC is addictive' while CBD is 'non-addictive' - I think there are many who would contest this statement. THC has largely been known as the cannabinoid that psychoactive, however there is extensive literature that maintains that cannabinoids are not addictive. If this is a statement that the authors hold, there should be a great discussion and engagement with why particular strains of cannabinoids are addictive. Giving more context to both the opposing effects on certain organ systems, as well as the debate over the different natures of CBD vs THC would help to better foreground the rationale for hypothesis i.

Response 3.1: Thank you. We have amended the word ‘addictive’ and instead referred to the intoxicating effects of THC while CBD itself is non-intoxicating.

Changes made to Introduction section, 2^nd^ paragraph, “THC can cause intoxication [4, 5] and has anti-emetic, analgesic and potentially neuroprotective and anti-inflammatory effects. On the other hand, CBD is non- intoxicating [5, 6]….”

We have also elaborated on the opposing effects of THC and CBD as suggested, and added to the 3^rd^ paragraph in the Introduction section:

“…., THC can cause intoxication and may induce anxiety and transient psychotomimetic effects [5], especially at higher doses and in vulnerable individuals, while CBD does not cause intoxication [5] and may potentially ameliorate anxiety and psychosis [9, 22-24]. Further, there is growing evidence that THC and CBD may have opposing acute effects on autonomic arousal, brain [15] and cardiovascular function [25, 26] and CBD may mitigate some of the harmful effects of THC on cognition and behaviour [15, 27, 28], consistent with their opposing effects on some of their molecular targets [4]. This suggests that THC and CBD may have distinct tolerability profiles, with the possibility that certain side-effects may be noticeable in those taking formulations containing only THC, but not in those taking formulations containing only CBD while they may even be mitigated in those taking THC and CBD in combination. This underscores the importance of examining their safety and tolerability separately……”

Comment 3.2: While it RCTs are privileged in meta-analysis reviews, it would be good if the authors could also make reference to the amount of literature that is being published such as open-label trials, n-of-1 studies, as well as observational studies where CBMs and their safety are being observed. While these methods certainly don't have the same level of rigour or control, it is worth noting, particularly in terms of how these studies align (or disagree) with the results presented in this review.
Response 3.2: Thank you. In response to this comment, we have now discussed our results in the context of evidence from observational studies (8^th^ Paragragh, under Discussion), which was originally present in the supplementary discussion:

“……These results are also consistent with a prospective observational study of 901 people above 65 years of age (74.5 ± 7.5 years), who received medical cannabis from January 2015 to October 2017 in a specialized medical cannabis clinic, 31.7% reported at least one AE due to the treatment after six months, with dizziness (9.7%) and dry mouth (7.1%) as the commonest AEs [21]. Another similar study of 184 people (81.2 ± 7.5 years of age) from April 2017 to October 2018 showed 33.6% with AEs and dizziness (12.1%) and sleepiness and fatigue (11.2%) as the commonest AEs [91].”

Comment 3.3: On page 6, it would be useful to give a brief overview of the broader subgroups of disease conditions. Also, if possible and to provide context, it would be useful at some point in the article to outline what the common treatments (particularly pharmaceutical) are used for these conditions, and whether treatment side effects differ greatly compared to those reported in this review.

Response 3.3: Thank you. In response to this comment, we have now referred to the broad subgroups of disease conditions and the common categories pharmacological treatments for these conditions in the Results section, 2^nd^ and 3^rd^ paragraph,

“The formulations used in THC studies were (numbers within brackets indicating the number of RCTs where each formulation was used): nabilone (6), dronabinol (marinol) (14), THC (3), THC extract spray (2) and Namisol (5). The combination THC:CBD trials used THC:CBD spray (18), cannabis extract (6) and CBD/THCV (2). The CBD studies used CBD preparations only.

The disease conditions investigated were classified into broader subgroups for analysis purpose as neurodegenerative (Alzheimer’s disease, Parkinson’s disease, Huntington’s disease. Amyotrophic lateral sclerosis), multiple sclerosis, motor neuron disease, pain (neuropathic pain), cancer (cancer or chemotherapy related anorexia, pain or nausea/vomiting), other (type 2 diabetes mellitus, chronic obstructive pulmonary disease, fibromyalgia, raised intraocular pressure, cervical dystonia, healthy, pancreatitis, obstructive sleep apnoea and Levodopa induced dyskinesia in Parkinson’s disease)”.

We believe that a systematic comparison of side-effect profile of CBM and these other treatments is outside the scope of the present review. Nevertheless, in response to this comment, we have amended the discussion, 13^th^ paragraph,

“Results presented here may also need to be considered against the side-effect profile of common treatments for the clinical conditions investigated in studies included in the meta-analyses reported here. They included patients with Alzheimer’s disease, Parkinson’s disease, Huntington’s disease. Amyotrophic lateral sclerosis, multiple sclerosis, motor neuron disease, neuropathic pain, cancer (cancer or chemotherapy related anorexia, pain or nausea/vomiting), type 2 diabetes mellitus, chronic obstructive pulmonary disease, fibromyalgia, raised intraocular pressure, cervical dystonia, pancreatitis and obstructive sleep apnoea. As would be expected, these conditions are typically treated with a wide range of pharmacological treatments (opioid analgesics, non-steroidal anti-inflammatory, anti-epileptics, benzodiazepines, psychotropics, cholinesterase inhibitors, glutamate antagonists, dopamine agonists, immunosuppressants, muscle relaxants, etc). While a systematic comparison of side-effect profile of CBMs and these other treatments would be useful and can helpfully inform prescribing decisions, this was outside the scope of the present review and was not examined here. Therefore, future studies may need to investigate this in a systematic manner.”

Comment 3.4: It would be useful at the beginning of the discussion to re-state the hypotheses, and more clearly align the findings of the review to each of them. As noted, there were some findings that were contrary to predictions, and while there is some speculation for the cause of this difference, more literature could be drawn on to make sense of the finding.

Response 3.4: Response We have now amended the 1^st^ paragraph in Discussion section:

“In this systematic review and meta-analysis, we investigated the safety and tolerability of medicinal cannabinoids in older adults by pooling data from double-blind RCTs with a reported mean participant age of 50 years and over. We predicted that compared to control treatments all 3 categories of CBMs will be associated with a greater incidence of AEs, but no greater incidence of SAEs, death or risk of withdrawal from study. We also expected a direct relationship between the dose of THC used in THC-containing CBMs and the risk of adverse outcomes…………

…. In terms of relationship between THC dose and adverse outcomes, as predicted, we found a direct relationship between daily THC dose and all cause AEs and AE-related withdrawals in THC studies and between THC dose and AE-related withdrawals in THC:CBD studies. Contrary to our predictions, we did not find any association between THC dose and the other outcomes investigated, such as SAEs and deaths.”

4^th^ paragraph in Discussion section:

“... Although we did observe a significant association between THC dose and all cause AEs in THC studies, this was present in THC:CBD studies. Whether this reflects independent evidence that CBD may mitigate some of the adverse effects of THC, such as on cognition, behaviour, autonomic and cardiovascular function [15, 25-28] and therefore may have obscured any dose-response relationship with AEs in THC:CBD studies remains unclear. The present study was not designed to formally test this hypothesis, which needs independent examination.”

We have now moved the supplemental discussion to the main manuscript (paragraphs 7-9 under Discussion section) to discuss the differences;

“As in previous reviews, we found that not all studies have published all the AEs and SAEs and that there is lack of evidence of the safety, tolerability and acceptability specifically in older patients. Nevertheless, our findings are consistent with previous reviews which found dizziness to be the most common non-serious AE with CBM treatment [12, 16, 17]. These results are also consistent with a prospective observational study of 901 people above 65 years of age (74.5 ± 7.5 years), who received medical cannabis from January 2015 to October 2017 in a specialized medical cannabis clinic, 31.7% reported at least one AE due to the treatment after six months, with dizziness (9.7%) and dry mouth (7.1%) as the commonest AEs [21]. Another similar study of 184 people (81.2 ± 7.5 years of age) from April 2017 to October 2018 showed 33.6% with AEs and dizziness (12.1%) and sleepiness and fatigue (11.2%) as the commonest AEs [91].

A previous systematic review of the efficacy and safety of medicinal cannabinoids which focused only on older people considered 5 controlled trials with THC (n = 3) and oral THC:CBD (n = 2)[12]. However, this review did not provide summary estimates (effect sizes) due to high heterogeneity among the included studies, lack of available data on means and standard deviations per treatment group, and very small sample sizes[12]. Therefore, only qualitative and descriptive summaries were provided. Studies included in this review reported dizziness, euphoria, drowsiness, confusion, and disorientation as the common adverse effects [12], consistent with our report. An earlier systematic review of AEs of medicinal cannabinoids for all ages by Wang et al [17] included 23 RCTs published between 1966 and late 2007, and analysed the effect of oral THC, and THC:CBD oral and spray formulations. However, they did not include data from studies examining the synthetic cannabinoid nabilone and found no evidence of higher incidence of SAEs after a median of 2 weeks use compared with a control group, regardless of the age of individuals (RR 1.04, 95% CI 0.78 -1.30). Wang and colleagues[17] reported that respiratory, gastrointestinal and nervous system disorders were the most frequently reported categories of SAEs and dizziness was the most commonly reported nonserious AE (15.5%) among people exposed to cannabinoids. However, unlike the present review wherein we have used GRADE criteria to assess methodological quality of included RCTs, Wang et al used a scale reported in a manuscript by Jadad and colleagues[92] to assess methodological quality of RCTs. As the scale from Jadad and colleagues does not adequately assess the quality of safety reporting in RCTs, most studies were rated as of good quality in the review by Wang et al, despite their poor reporting of safety[17].

However, our results are not consistent with a more recent systematic review and meta-analysis by Whiting et al[16], which included studies investigating all age groups and showed that SAEs and AE-related withdrawal are generally higher in those treated with CBMs. In contrast, we have shown that SAEs are not increased in older adults consistent with other reviews[12, 17]. Whiting et al[16] found pooled effect-size for any AE (OR 3.03,95% CI 2.42-3.80), SAE (OR 1.41, 95% CI 1.04-1.92) and withdrawal due to AE (OR 2.94, 95% CI 2.18-3.96) and Wang et al[17] found significantly higher non-serious AEs (RR 1.86, 95% CI 1.57-2.21) in those who received CBMs compared to control groups. In contrast to Whiting et al, we have shown that increase in withdrawal may be more nuanced and more in those receiving THC:CBD combination and not for other types of CBMs.”

Comment 3.5: There are a number of sentences throughout that read as incomplete or require a re-write that could be part of the revision process.

Response 3.5: Apologies for this oversight. We have now amended these.

Again, we appreciate all of your insightful comments. We tried to address each of your points in detail. We feel the revision represents an improvement and hope you do also. Thank you for taking the time to help us improve our paper.

References:

1, Higgins JP, Thompson SG, Deeks JJ, Altman DG. Measuring inconsistency in meta-analyses. BMJ. 2003 Sep 6;327(7414):557-60. doi: 10.1136/bmj.327.7414.557.

2. von Hippel PT. The heterogeneity statistic I(2) can be biased in small meta-analyses. BMC Med Res Methodol. 2015 Apr 14;15:35. doi:10.1186/s12874-015-0024-z.
