# Supplementary material for: Safety and tolerability of natural and synthetic cannabinoids in adults aged over 50 years: A systematic review and meta-analysis
Source: PLoS Med. 2021 Mar 29;18(3):e1003524. doi: 10.1371/journal.pmed.1003524 (PMC8007034; doi:10.1371/journal.pmed.1003524)
Supplement: Attachment — Submitted filename: Author Response_revision 2.docx [file pmed.1003524.s004.docx]

To	10.12.2020

Prof Clare Stone

Editor-in-Chief

PLOS Medicine

Dear Editor-in-Chief

Ref: Manuscript Reference Number: **PMEDICINE-D-20-04864R2**

Title: **Safety and tolerability of natural and synthetic cannabinoids in adults aged over 50 years: a systematic review and meta-analysis**

Thank you very much for reviewing the manuscript and for the valuable comments to further improve our paper. Please find attached the revised manuscript for re-submission.

Detailed explanations about our revisions are included in the following RESPONSE TO REVIEWERS section. We hope that you will find that our responses to comments from the editorial team have adequately addressed the issues raised.

We look forward to hearing from you in due course.

Sincerely yours,

Sagnik Bhattacharyya

RESPONSE TO REVIEWERS:

Requests from the editors:

Editor1.1. Were there any departures from the study protocol? 
Response: In our PROSPERO protocol, we had mentioned only about STROBE guidelines for assessment of study quality for observational studies, but had not mentioned GRADE criteria, which are appropriate for RCTs. Therefore, we have used GRADE to evaluate the quality and risk of bias of RCTs synthesized in the present manuscript. Further, we carried out an updated search in response to reviewer’s comments. There were no other departures from the study protocol.

Editor1.2. Please consider adapting the title from "... older adults" to "... adults aged over 50 years". 
Response: We have now amended the title as suggested.

Editor1.3. We would suggest also specifying the relevant age range in the first subsection of your abstract. 
Response: We have now added this information in the abstract (Para 1):

“We aimed to conduct a systematic review and meta- analysis of safety and tolerability of cannabinoid-based medicines (CBMs) in adults of age ≥50 years……..”

Editor1.4. Please make that "data were" in your abstract.
Response: We have now amended this.

Editor1.5. Please adapt your abstract so that the summary of limitations is restricted to a single sentence, beginning "Study limitations include ..." or similar and falling at the end of the "Methods and findings" subsection. Please do not include mitigating arguments in this sentence. 
Response: We have now amended this section (Page 3, Para 1) in accordance with this comment and also removed mitigating arguments:

“Study limitations include inability to fully exclude data from those < 50 years age in our primary analyses as well as limitations related to weaknesses in the included trials particularly incomplete reporting of outcomes and heterogeneity in included studies….”

Editor1.6. Please adapt the author summary so that one or two items use the active voice, e.g. "We analysed pooled data ...". Please address "that that".

Response: Thank you. We have now amended the summary in accordance with these comments and have also removed the extra ‘that’ (apologies for this oversight). The amended sentences read as:

Page 4, Para 3: “We analysed data on safety and tolerability from previously published double-blind, randomized controlled trials (RCT) using delta-9-tetrahydorcannabinol (THC) and cannabidiol (CBD) the common constituents of most CBMs, alone or in combination, to examine their effect on older adults.”

Page 4, Para 4: “We pooled data from 46 published RCTs (with information from 6216 patients; with mean participant age ≥ 50 years) on adverse events, serious adverse events or death, and withdrawal from study. We also examined the relationship between the dose of THC used in THC-containing CBMs and the incidence of adverse consequences in older adults.”

Editor1.7. Please trim and reorganize the author summary so that it consists of up to 3 points per subsection, with each point consisting of no more than 1-2 short sentences. 
Response: We have now amended the summary thoroughly in accordance with this comment.

Editor1.8. Early in the Discussion section, please adapt "We predicted ..." to "We hypothesized ..." or similar. You mention "predictions" elsewhere in the same section of the ms, and in your Introduction, and we suggest rewording here too.

Response: Thank you. In accordance with this comment, we have amended “predicted” to “hypothesized” and “predictions” to “hypotheses” or “expectations” throughout the Introduction and Discussion sections. We have not made any changes wherever “prediction’ has come up in the context of ‘prediction interval” or “predicted” effects across different contexts.

Editor1.9. Please revisit "gender", used in the Discussion section and perhaps elsewhere, substituting "sex" where appropriate.

Response: Done (this was only once in the Discussion section, Page 35, Para 2).

Editor1.10. Noting references 30 & 67, for example, please ensure that information on competing interests and so on is removed from all relevant citations.

Response: Thank you. We have removed these now.

Editor1.11. Please adapt the attached PRISMA checklist so that individual items are referred to by section (e.g., "Methods") and paragraph number rather than by line or page numbers, as the latter generally change in the event of publication. Please refer to the checklist early in the methods section of your main text ("See S1_PRISMA_Checklist" or similar).

Response: The PRISMA checklist has been amended as suggested and is now referred to early on in the Methods section of the main text (Page 8, Para 2, shown below). The original “S1 Appendix” has been re-labelled as “S2 Appendix” and reference to it throughout the text has been amended to reflect this.

“The review was undertaken according to the Preferred Reporting Items for Systematic Reviews and Meta-analyses (PRISMA) reporting guidelines [29] (see S1 PRISMA checklist)….”

Editor 1.12. Please adapt the title of fig 1 to "Study disposition" or similar.

Response : The title has been amended to “Study disposition”.

Editor 1.13. RevMan permitting, please avoid "p=0.000" in fig 2a.

Response : This figure has been amended.

Editor 1.14. Please remove the draft paper included as an attachment.

Response: Done.

Comments from Reviewers:

*** Reviewer #1: 

Reviewer 1.1. The authors have addressed my concerns and I now recommend publication.
Response: Thank You.

*** Reviewer #2:

Reviewer 2.1. Excellent revision of manscript. No more comments!

Response: Thank You.
